# The Role of Glial Fibrillary Acidic Protein as a Biomarker in Multiple Sclerosis and Neuromyelitis Optica Spectrum Disorder: A Systematic Review and Meta-Analysis

**DOI:** 10.3390/medicina60071050

**Published:** 2024-06-26

**Authors:** Aysa Shaygannejad, Nazanin Rafiei, Saeed Vaheb, Mohammad Yazdan Panah, Vahid Shaygannejad, Omid Mirmosayyeb

**Affiliations:** 1 Isfahan Neurosciences Research Center, Isfahan University of Medical Sciences, Isfahan 81839-83434, Iran; aysash2001@gmail.com (A.S.); saeedvaheb.sv@gmail.com (S.V.); v.shaygannejad@gmail.com (V.S.); 2 School of Medicine, Isfahan University of Medical Sciences, Isfahan 81746-73461, Iran; nazaninrafiei21@gmail.com; 3 Student Research Committee, Shahrekord University of Medical Sciences, Shahrekord 88157-13471, Iran; mohamad.yazdanpanahh@gmail.com; 4 Department of Neurology, Isfahan University of Medical Sciences, Isfahan 81746-73461, Iran

**Keywords:** glial fibrillary acidic protein, multiple sclerosis, neuromyelitis optica spectrum disorder

## Abstract

There is debate on the role of glial fibrillary acidic protein (GFAP) as a reliable biomarker in multiple sclerosis (MS) and neuromyelitis optica spectrum disorder (NMOSD), and its potential to reflect disease progression. This review aimed to investigate the role of GFAP in MS and NMOSD. A systematic search of electronic databases, including PubMed, Embase, Scopus, and Web of Sciences, was conducted up to 20 December 2023 to identify studies that measured GFAP levels in people with MS (PwMS) and people with NMOSD (PwNMOSD). R software version 4.3.3. with the random-effect model was used to pool the effect size with its 95% confidence interval (CI). Of 4109 studies, 49 studies met our inclusion criteria encompassing 3491 PwMS, 849 PwNMOSD, and 1046 healthy controls (HCs). The analyses indicated that the cerebrospinal fluid level of GFAP (cGFAP) and serum level of GFAP (sGFAP) were significantly higher in PwMS than HCs (SMD = 0.7, 95% CI: 0.54 to 0.86, *p* < 0.001, I^2^ = 29%, and SMD = 0.54, 95% CI: 0.1 to 0.99, *p* = 0.02, I^2^ = 90%, respectively). The sGFAP was significantly higher in PwNMOSD than in HCs (SMD = 0.9, 95% CI: 0.73 to 1.07, *p* < 0.001, I^2^ = 10%). Among PwMS, the Expanded Disability Status Scale (EDSS) exhibited significant correlations with cGFAP (r = 0.43, 95% CI: 0.26 to 0.59, *p* < 0.001, I^2^ = 91%) and sGFAP (r = 0.36, 95% CI: 0.23 to 0.49, *p* < 0.001, I^2^ = 78%). Regarding that GFAP is increased in MS and NMOSD and has correlations with disease features, it can be a potential biomarker in MS and NMOSD and indicate the disease progression and disability in these disorders.

## 1. Introduction

Multiple sclerosis (MS) is a chronic demyelinating autoimmune disease of the central nervous system (CNS) characterized by focal lesions in the gray and white matter [1,2]. There are approximately 2.8 million MS cases worldwide, with females being twice as likely to have the disease [3]. Neuromyelitis optica spectrum disorder (NMOSD) is an immunoglobulin (Ig) G antibody-mediated autoimmune disease mostly characterized by clinical features such as optic neuritis and myelitis [4,5]. NMOSD is considered a rare disease worldwide [6,7] which occurs mostly in females and younger patients aged between 30–40 [8].

There are several biomarkers to predict disease activity and progression in MS and NMOSD. Cerebrospinal fluid (CSF) levels of glial fibrillary acidic protein (GFAP), neurofilament light chain (NfL), myelin basic protein, and IgG-index are some of the biomarkers reported to be higher in people with MS (PwMS) [9,10,11]. GFAP is a type III intermediate filament constituting the cytoskeletal structure of the astrocytes in the CNS [12]. When astrocytes sustain damage, due to trauma or disease, GFAP is released into the CSF [13]. Consequently, disruption of the blood–brain barrier (BBB) can cause the entrance of GFAP into the bloodstream [13]. CSF and serum levels of GFAP (cGFAP and sGFAP) could serve as potential detecting biomarkers in patients with neurological disorders affecting astrocytes, such as MS and NMOSD [14,15].

GFAP has been reported to be increased in the magnetic resonance imaging (MRI) plaques of PwMS following damage to the astrocytes [16,17], and it has been found that higher cGFAP is associated with more disease progression and disability [18]. Given that NMOSD is classified as an astrocytopathy, cGFAP and sGFAP serve as effective biomarkers for assessing the activity and severity of NMOSD [19]. Some studies have also reported higher cGFAP and sGFAP in PwNMOSD compared to PwMS [20,21]. However, in some phenotypes of NMOSD that are seronegative for AQP4-IgG and MOG-IgG, the sGFAP has been reported to be much lower than those in the AQP4-IgG positive patients [22].

Due to the previous evidence and lack of a systematic review and meta-analysis to comprehensively assess the role of GFAP in MS and NMOSD, this review aimed to compare the level of GFAP between PwMS, PwNMSOD, and healthy controls (HCs), as well as the relationships of GFAP with disease activity and neurological disability in MS and NMOSD.

## 2. Methods

This study was conducted based on the preferred reporting items for systematic reviews and meta-analyses (PRISMA) guidelines [23]. These guidelines ensure a comprehensive and transparent approach to reviewing and synthesizing data, facilitating the rigorous and methodical evaluation required for our systematic review and meta-analysis.

### 2.1. Search Strategy

We comprehensively searched the following databases: PubMed, Embase, Scopus, and Web of Science, up to 20 December 2023. The search strategy incorporated MeSH terms and keywords relevant to multiple sclerosis, neuromyelitis optica spectrum disorders, and glial fibrillary acid protein, tailored for each database. More details of the search strategy are provided in Supplementary S1.

### 2.2. Study Selection

Two authors (SV and AS) independently screened the studies using a two-step process. First, the title and abstract of articles identified from the literature search were reviewed, and irrelevant articles were excluded. Then, based on the predominant inclusion and exclusion criteria, the full texts of the articles were assessed for eligibility and eligible papers were selected. The reference list of included studies and related reviews was manually reviewed to ensure the comprehensive inclusion of relevant studies. Any disagreements were resolved through consultation with a senior reviewer (OM).

### 2.3. Eligibility Criteria

Studies that met the following criteria were included:

(A) Published in English;
(B) Peer-reviewed original studies, including case-controls, cohorts, and cross-sectional studies;
(C) The study population consisted of adult people (age above 18 years) with confirmed diagnosis of MS or NMOSD;
(D) Either a report of cGFAP/sGFAP or a report of the correlations between cGFAP/sGFAP with demographic, clinical, or imaging findings.

Studies were excluded if they met the following criteria:

(A) Non-English studies;
(B) Case reports, case series, conference abstracts, and review articles;
(C) In vitro and animal studies;
(D) Lack of sufficient information on key elements.

### 2.4. Data Extraction

Two researchers (MYP and SV) independently extracted the following data from the included studies: author, country and year of publication, study design, sample size, demographics, MS type, disease duration, EDSS, assay type for GFAP, and features of MRI devices. Data extraction was carried out diligently and meticulously to ensure the utmost precision in our findings.

### 2.5. Risk of Bias Assessment

The Newcastle–Ottawa Scale (NOS) [24] was used to assess the quality of the studies included in our analysis, including the selection of the participants, comparability of study groups, and outcome assessment, with a score ranging from 0 to 9.

To ensure an unbiased evaluation, the quality of the included studies was independently assessed using NOS by two authors (MYP and NR). Any disagreement was resolved by a third researcher (OM).

### 2.6. Data Analysis

The meta-analysis was conducted on two distinct effect sizes. Initially, the pooled standard mean difference (SMD) and its 95% confidence interval (CI) for GFAP level were calculated between MS, NMOSD, and HC using Cohen’s d [25]. Cohen’s standardized SMD represents four levels of strength of effect sizes: no effect (SMD 0), small (SMD 0.2–0.4), medium (SMD 0.4–0.7), and large (SMD > 0.8) [26]. Then, a meta-analysis was conducted to determine the pooled correlation coefficients between GFAP level and demographic and clinical characteristics of patients with MS and NMOSD. In this analysis, the correlations were initially converted into Fisher’s z-scores. Subsequently, these z-scores were retransformed into correlation coefficients to facilitate their visualization and interpretation [27]. The correlation coefficient strength was categorized as follows: 0.00–0.10 as negligible, 0.10–0.39 as weak, 0.40–0.69 as moderate, 0.70–0.89 as strong, and 0.90–1.00 as very strong [28].

All statistical analysis was performed using R software version 4.3.3 with the “meta” package. Results were pooled and displayed in forest plots when three or more comparative studies reported the effect sizes. Given the potential methodological heterogeneity among the included studies, the random-effects model was utilized to conduct the meta-analyses. Subgroup analysis was conducted based on the sample source of GFAP (CSF or serum) when sufficient data regarding its origin was available. Heterogeneity among the included studies was evaluated using Cochran’s Q test and the inconsistency index [29]. The sensitivity analysis, employing the leave-one-out method, was utilized to assess the individual contribution or weight of each study to the overall effect of each meta-analysis [30]. Furthermore, the risk of publication bias was evaluated by visually inspecting funnel plots [31] and conducting Egger’s and Begg’s tests [32,33]. The statistical significance of all meta-analyses was considered as *p*-value less than 0.05.

## 3. Results

### 3.1. Literature Search and Study Selection

**Figure 1:**
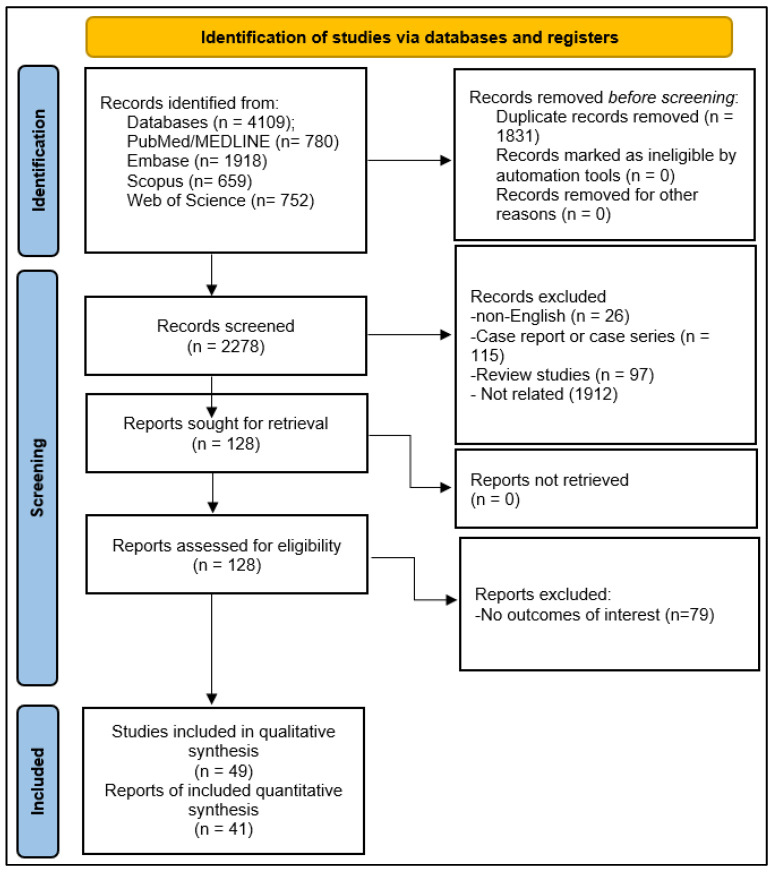
PRISMA flow diagram depicting the procedures of screening and study selection.

Our literature search across the databases yielded 4109 articles. After removing the duplicates, 2278 articles were selected for screening the titles and abstracts. During the screening, and after eliminating the articles based on the inclusion and exclusion criteria, the full texts of 128 remaining articles were obtained to critically assess the eligibility. Following the disqualification of the articles with insufficient data, 49 studies consisting of 3491 PwMS, 849 PwNMOSD, and 1046 HCs were enrolled for the qualitative and 41 studies for quantitative synthesis (Figure 1).

### 3.2. Characteristics of the Included Studies

This review included 49 studies involving 3491 PwMS, 849 NMOSD patients, and 1046 HCs. In 16 studies, GFAP level was measured in CSF [11,15,18,34,35,36,37,38,39,40,41,42,43,44,45,46]; however, it was measured in serum samples in 27 studies [19,20,47,48,49,50,51,52,53,54,55,56,57,58,59,60,61,62,63,64,65,66,67,68,69,70,71]. Six studies measured it in both CSF and serum samples [14,72,73,74,75,76]. The included studies were published within the timeframe from 2002 to 2023. PwMS (*n* = 3491) demonstrated a mean (SD) age of 43.6 (12.5) years, a disease duration of 10.4 (12.2) years, and an EDSS score of 3.1 (2.1), with 65.7% of them being female. Among NMOSD patients (*n* = 849), 87.9% were female, with an average (SD) age of 44.4 (14.9) years, disease duration of 6.4 (15.4) years, and EDSS scores of 3.6 (2.2). The overview of the principal characteristics of the included studies is summarized in Table 1.

### 3.3. Outcomes Synthesis

#### 3.3.1. Comparison of the GFAP Level between MS and HCs

**Figure 2:**
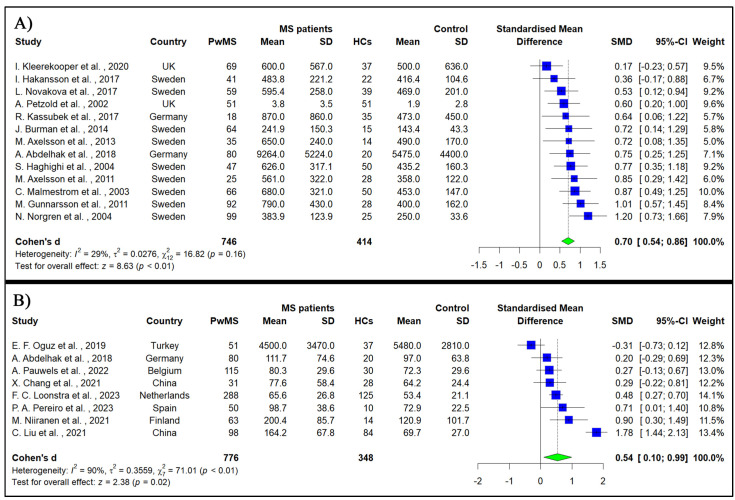
Forest plot of the meta-analysis of pooled standard mean difference of glial fibrillary acidic protein level between people with multiple sclerosis and healthy controls: (**A**) cerebrospinal fluid sample [11,14,15,18,36,37,38,39,41,42,44,45,46], (**B**) serum sample [14,49,54,57,63,64,66,71].

A meta-analysis of thirteen studies assessing the cGFAP of 746 PwMS and 414 HCs demonstrated a statistically significant elevation in GFAP among PwMS compared to HCs (SMD = 0.7, 95% CI: 0.54 to 0.86, *p*-value < 0.001, I^2^ = 29%) (Figure 2A).

A meta-analysis of eight studies evaluating sGFAP in 776 PwMS and 348 HCs revealed a statistically significant increase in sGFAP in PwMS compared to in the HCs (SMD = 0.54, 95% CI: 0.1 to 0.99, *p*-value = 0.02, I^2^ = 90%) (Figure 2B).

#### 3.3.2. Comparison of the GFAP level between PMS and RRMS

The meta-analysis of seven studies investigating cGFAP of 199 progressive MS (PMS) and 267 relapsing–remitting MS (RRMS) patients indicated that PMS patients had a significantly higher cGFAP than RRMS patients (SMD = 0.45, 95% CI: 0.22 to 0.69, *p*-value < 0.001, I^2^ = 34%) (Table 2).

According to the meta-analysis of six studies measuring the sGFAP level of 265 PMS and 490 RRMS patients, a significantly increased sGFAP was found in PMS patients compared to in RRMS patients (SMD = 0.5, 95% CI: 0.25 to 0.75, *p*-value < 0.001, I^2^ = 53%) (Table 2).

#### 3.3.3. Comparison of the GFAP Level between NMOSD and HCs

**Figure 3:**
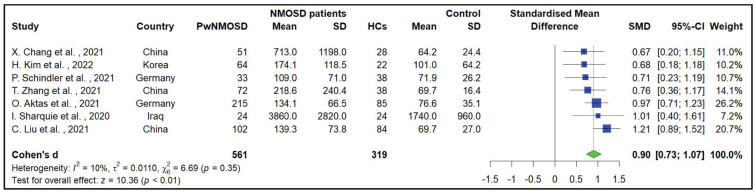
Forest plot of the meta-analysis of pooled standard mean difference of serum level of glial fibrillary acidic protein between people with neuromyelitis optica spectrum disorder and healthy controls [13,19,60,61,64,66,69].

Based on the meta-analysis of seven studies encompassing 561 NMOSD patients and 319 HCs, the sGFAP was significantly higher in PwNMOSD than in HCs (SMD = 0.9, 95% CI: 0.73 to 1.07, *p*-value < 0.001, I^2^ = 10%) (Figure 3).

Comprehensive results of meta-analysis of SMD are provided in Table 2 and Supplementary S2.

#### 3.3.4. Correlation Coefficients between GFAP Level and Demographic, Serologic, Imaging, and Clinical Findings of PwMS

Among PwMS, the sGFAP exhibited the most significant correlations with Nfl (r = 0.42, 95% CI: 0.32 to 0.52, *p*-value < 0.001, I^2^ = 76%), T2 lesion volume (T2LV) (r = 0.37, 95% CI: 0.29 to 0.46, *p*-value < 0.001, I^2^ = 0%), EDSS (r = 0.36, 95% CI: 0.23 to 0.49, *p*-value < 0.001, I^2^ = 78%), and disease duration (r = 0.28, 95% CI: 0.15 to 0.41, *p*-value < 0.001, I^2^ = 53%) respectively. Additionally, the cGFAP had significant relationships with EDSS (r = 0.43, 95% CI: 0.26 to 0.59, *p*-value < 0.001, I^2^ = 91%) and Nfl (r = 0.39, 95% CI: 0.29 to 0.49, *p*-value < 0.001, I^2^ = 38%). Further details of the meta-analysis on correlation coefficients are summarized in Table 3.

### 3.4. Sensitivity Analysis

The sensitivity analysis detected no outliers or points of significant influence in any of the meta-analyses. The sensitivity analysis results are detailed in the Supplementary S2.

### 3.5. Publication Bias

According to the funnel plots apparent, and by statistical findings from Begg’s and Egger’s tests, there was no indication of publication bias in any of the meta-analyses. The funnel plots and the statistical outcomes derived from Begg’s and Egger’s tests for all conducted analyses are presented in Table 2 and Table 3 and Supplementary S2.

### 3.6. Risk of Bias Assessment

Of the 53 eligible studies subject to ROB assessment, 34 garnered ratings surpassing six stars, while 15 fell within the range of from 4 to 6 stars. The mean (SD) of the ROB assessment score across the studies was 6.9 (1.2), indicating moderate to high ratings on the NOS for constitute studies (Table 1).

## 4. Discussion

The current study clearly illustrated the heightened level of GFAP in MS and NMOSD compared to HCs, indicating that GFAP can be a potential biomarker in MS and NMOSD. Additionally, PMS patients had higher GFAP levels compared to those with RRMS, highlighting its utility in identifying more severe disease states. Furthermore, the GFAP level exhibited relationships with some clinical characteristics, serological biomarkers, and imaging measures of PwMS and PwNMOSD. These findings imply the potential role of GFAP in MS and NMOSD. Several studies have reported increased cGFAP and sGFAP in PwMS [11,14] and PwNMOSD [19,77], further supporting our results [14,70,78,79].

Astrocytes compromise most CNS cells [80], providing functional and structural support for neurons [81]. These glial cells consist of GFAP, an intermediate filament III protein, which is also expressed in non-myelinating Schwann cells in the peripheral nervous system and the enteric glial cells of the enteric nervous system [81,82]. GFAP plays a role in the motility and morphology of astrocytes, as well as the cellular functioning of the BBB [83]. When under stressful conditions, such as CNS trauma/disease, astrocytes react through reactive astrogliosis where proliferation, hypertrophy, and increased protein, such as GFAP, expression happens [12]. The activation of astrocytes leads to morphological changes, such as the hypertrophy of cell bodies and retraction of astrocytic end-feet, which leads to BBB disruption, allowing the entry of inflammatory factors [84]. Decreased homeostatic functions also accompany the activation process [85].

The gliosis of the astrocytes refers to the formation of these cells as a protective barrier surrounding the scar tissue at the center of the lesion in the damaged area [86]. Interestingly, certain levels of astrogliosis seemed beneficial for neuroprotection and post-injury recovery, while excessive gliosis associated with neuroinflammation has the opposite effect on the structural and functional recovery of the CNS [87,88].

Following the neuroinflammation and astrogliosis in the damaged areas, an increase in GFAP level is seen [89]. Elevation in the GFAP protein level is a distinguished feature of degenerative diseases [81] such as MS and NMOSD. It is the principal protein found in chronic lesions of MS and is released in CSF from degenerating brain cells [90]. GFAP can also be found in the peripheral blood following damage to the BBB [91]. Hence, the presence of GFAP in the blood may indicate CNS injury.

Astrocytic activation initiates at an early stage of MS, persists into the chronic phase of the disease, and resumes even after the absence of immune cells [84,88]. Autopsies have shown higher levels of GFAP in the cortices of PwMS than those observed in HCs [18]. Elevated cGFAP indicates astrocyte activation, a hallmark of neuroinflammation [34]. Astrocytes, when activated in MS, may enhance neurodegenerative pathways and are linked to the progression of disability in PwMS [34]. It was found that cGFAP correlated with inflammatory cytokines and was associated with an increased risk of disease progression in RRMS [34]. Furthermore, GFAP has been investigated in the context of Parkinson’s disease as a biomarker of disease progression [92]. GFAP level has been found to correlate with other key biomarkers, providing insights into the neurodegenerative process and offering the potential for monitoring disease advancement over time [92].

PMS patients has been reported to have elevated cGFAP than RRMS patients, indicating that this protein may be a marker for disease progression [93]. Abdelhak et al. suggested that the increased activation of astrocytes in advanced stages of MS compared with early stages, leading to higher GFAP release, might be responsible for this difference [14]. They also propose the GFAP to NfL ratio, which they found higher in PMS patients [14]. As NfL is an established marker for neuroaxonal damage, it was explained that axonal damage is displayed more in active lesions, which are predominant in RRMS brains, than in chronic-active or inactive ones in PMS [94,95]. Therefore, these higher levels of GFAP may also be explained by the type of lesions in PMS patients. Hogel et al. have suggested that sGFAP is associated with disease progression and could act as an early biomarker of progression in MS [96].

Serum autoantibodies against AQP4, a water channel protein on the perivascular end-feet processes of astrocytes, distinguish NMOSD from MS [97,98]. These autoantibodies lead to astrocyte destruction and, consequently, the release of astrocytic contents, including GFAP, into the CSF and serum [21,99]. Elevated cGFAP and sGFAP have been detected in PwNMOSD [44,100].

Previous studies have not sufficiently investigated the difference in serum and CSF levels of GFAP between PwMS and PwNMOSD [15,20,43,64,66,68,76]. Most prior research found higher GFAP levels in NMOSD than in MS [15,43,66,68,76]. However, two studies indicated different findings [20,64]. It was suggested that a higher level of GFAP in NMOSD than in MS and HCs may result in astrocyte destruction following AQP-4 antibody activity [66]. However, a definitive conclusion remains elusive, and further research is necessary to explore and compare GFAP levels between MS and NMOSD.

According to the meta-analyses, GFAP level was associated with EDSS, disease duration, Nfl, and T2LV in MS. Additionally, GFAP exhibited relationships with disability in NMOSD. Elevated cGFAP has been linked with early progression to disabilities in PwMS [101]. Hogel et al. have found elevated levels of both NfL and GFAP in PwMS to be associated with higher EDSS, longer disease duration, and MRI pathology, which agrees with our results [96]. Other studies have reported positive correlations between GFAP and T2LV [62,70]. Abdelhak et al. showed a strong correlation between sGFAP and EDSS. However, the result was applied only to patients with PMS, not RRMS [14]. They also found correlations between cGFAP, sGFAP, and NfL in the MS group, which were stronger in primary progressive MS (PPMS) patients [14]. There is a hypothesis that the rise in GFAP level is associated with more profound neuroaxonal damage and disease progression, which may explain the correlation between GFAP and EDSS [62]. NfL is a structural protein of the axonal cytoskeleton proposed as a useful neurodegenerative biomarker [102]. The positive correlation between GFAP and NfL shows the release of these proteins from damaged cells in the CNS throughout a degenerating disease such as MS. The relationships between GFAP and demographic, clinical, and neuroimaging features remain insufficiently defined in MS and NMOSD. Thus, further investigations are necessary to elucidate this domain.

Elevated levels of GFAP in MS and NMOSD may significantly refine patient care strategies [34]. As a biomarker, GFAP’s longitudinal tracking may offer clinicians a tool for assessing disease activity and progression, but more studies are required in this field. Such data could lead to proactive, individualized adjustments in therapy, possibly averting exacerbations and worsening of disability. The clinical application of GFAP level as a decision-making aid in treatment regimens underscores the move toward personalized medicine, emphasizing its potential as a harbinger of neuroinflammatory activity and a guide in optimizing therapeutic interventions.

## 5. Limitations and Strengths

While this systematic review and meta-analysis offers some insightful observations on using GFAP as a biomarker, some key limitations must be acknowledged. There was a mix of factors like disease severity, treatment backgrounds, and age across the studies, and these need to be consistently controlled in primary studies. This study does not delve into longitudinal GFAP levels over time either, which limits our ability to understand if or how GFAP tracks disease progression. Moreover, the lack of sufficient studies prevented us from comparing GFAP levels between MS and NMOSD groups.

To the best of our knowledge, this is the first systematic review and meta-analysis fully investigating the potential of GFAP as a biomarker in MS and NMOSD, as well as its association with clinical and demographical characteristics of the diseases. Furthermore, we conducted the meta-analysis on GFAP level in CSF and serum samples of PwMS and PwNMOSD separately.

## 6. Conclusions

In conclusion, this review revealed elevated serum and CSF levels of GFAP in MS and NMOSD compared to healthy populations. Additionally, GFAP exhibited associations with disease duration, disability, NfL, and T2LV in MS. These findings underscore the potential role of GFAP in MS and NMOSD and suggest that GFAP could be a potential biomarker for monitoring and evaluating disability and disease progression in MS. However, additional longitudinal studies are warranted to validate these findings and elucidate other aspects related to the role of GFAP in the clinical practice of MS and NMOSD.

## Figures and Tables

**Table 1:** Study overview; main characteristics of included studies.

First Author, Country, Year	Study Design	PwMS				PwNMOSD			Healthy Controls	Assay Type	MRI Strength MRI Device	Key Findings	QA
		Sample Size, F to M Ratio Age; Mean (SD)	MS Type (n)	Disease Duration (Years); Mean (SD)	EDSS	Sample Size, F to M Ratio, Age; Mean (SD)	Disease Duration (Years); Mean (SD)	EDSS	Sample Size, F to M Ratio, Age; Mean (SD)				
L. Midaglia Spain 2023 [47]	Cohort	80 3 34.1 (8.4)	RRMS: 80	4.8 (5.2)	2 (1.5–2.5) **	NR	-	-	NR	Serum ELISA	1.5T NR	MRI correlated with GFAP, and both have prognostic implications in treatment response and long-term disease outcomes.	7
J. Schaefer Germany 2023 [48]	Cross-sectional	102 2.8 36 (11.3)	RRMS: 76 SPMS: 8 PPMS: 4 CIS: 10 RIS: 2	NR	NR	2 NR	NR	NR	NR	Serum ELISA	3T Siemens	Biomarkers may help stratify the application of contrast agents for brain imaging in MS patients.	10
F. Loonstra Netherlands 2023 [49]	Case- control	288 2.5 53.1 (1)	RRMS: 171 SPMS: 79 PPMS: 37	12 (5.5–18.6) **	3.5 (2.5–4.5) **	NR	-	-	125 2.8 53 (1.2)	Serum ELISA	3T Milwaukee	This demonstrates the potential of sGFAP as a complementary biomarker of neurodegeneration, reflected by disability, in progressive MS.	8
Y. Li China 2023 [50]	Cohort	NR	-	-	-	15 14 43 (31.8–57.2) **	2.5 (1.5–3.9) **	4.5 (3.7–6.1) **	NR	Serum ELISA	3T General Electric	Found a trend for sGFAP level predicting spinal cord atrophy in patients with NMOSD.	6
D. Jakimovski USA 2023 [51]	Cohort	202 3 47.1 (11.1)	RRMS: 148 PMS: 54	13.4 (10.2)	2.5 (1.5–5) **	NR	-	-	NR	Serum ELISA	3T Milwaukee	Baseline serum GFAP level can predict future disability progression.	8
G. Bose USA 2023 [52]	Cohort	144 1.7 37.4 (29.4–45.4) **	NR	1.1 (0.7–1.5) **	1.2 (0–2) **	NR	-	-	NR	Serum ELISA	1.5T General Electric	Worse clinical outcomes, SPMS and EDSS, are associated with higher sGFAP level.	7
C. Barro Switzerland 2023 [53]	Cohort	257 1.9 49 (11.3)	PPMS: 22 PMS: 235	14.7 (10.5)	4 (1.2)	NR	-	-	NR	Serum ELISA	3T NR	sGFAP level may be used to stratify patients with progressive MS.	9
P. Pereiro Spain 2023 [54]	Case- control	50 1.8 36.6 (9)	RRMS: 50	20.4 (18–23.5) **	2 (1.5–7.5) **	NR	-	-	10 1 40.5	Serum ELISA	NR	sGFAP level demonstrated a lower or no ability to differentiate between the long-term outcomes of RRMS.	7
A. Abdelhak Germany 2023 [55]	Cohort	243 1.2 55.5 (49.7–61.2) **	PPMS: 135 SPMS: 108	12 (6–21)**	4.5 (3.5–6)**	NR	-	-	NR	Serum ELISA	NR	A high GFAP level could distinguish non-active pwPMS with particularly high progression risk.	8
S. Thebault Canada 2022 [56]	Cohort	58 1.3 37.7 (6.7)	RRMS: 32 SPMS: 14 PPMS: 12	6.2 (3)	4 (2.5)	NR	-	-	NR	Serum ELISA	1.5T NR	Both baseline and longitudinal change in GFAP may help identify patients who would benefit from early treatment.	6
A. Pauwels Belgium 2022 [57]	Case- control	115 1.7 47 (13)	RRMS: 87 PPMS: 28	12 (14)	3 (3)	NR	-	-	30 1.7 52.5 (13.7)	Serum ELISA	NR	Both pGFAP and pNfL were related to worsening in PwMS.	7
F. Azzolini Italy 2022 [34]	Cross-sectional	51 2 36.5 (27.3–45.3) **	RRMS: 51	5 (1.7–29) **	1.5 (1–2) **	NR	-	-	NR	CSF ELISA	3T Milwaukee	Expression of CSF GFAP may characterize patients with a higher risk of progression.	7
H. Kim South Korea 2022 [58]	Case- control	NR	-	-	-	64 9.6 51 (45–60) **	6.7 (2–12.3) **	3 (2–4) **	22 3.4 51 (33–63) **	Serum ELISA	NR	sGFAP might be the most appropriate for monitoring NMOSD longitudinally, which warrants future confirming studies.	8
L. Aly Germany 2022 [59]	Case- control	21 3.2 38 (11.4)	RRMS: 21	5.6 (4.1)	1.4 (1.2)	16 4.3 46.6 (10)	6.1 (2.6)	3.4 (2.4)	21 3.2 42 (9.5)	Serum ELISA	NR	sGFAP have been introduced as new biomarkers for disease activity and disability in RRMS and NMOSD.	7
T. Zhang China 2021 [60]	Case- control	NR	-	-	-	72 8 49 (33.3–59) **	2.7 (1.8–7.2) **	3.3 (2–7) **	38 5.3 41 (29.8–55.3) **	Serum ELISA	NR	pGFAP may serve as a biomarker for NMOSD disease activity and treatment effects.	7
P. Schindler Germany 2021 [61]	Case- control	NR	-	-	-	33 10 50 (14)	6.5 (4.3–9) **	4 (2–5) **	38 4.4 42 (13)	Serum ELISA	NR	sGFAP has a potential role in disease severity and future disease activity in patients with NMOSD	6
M. Saraste Finland 2021 [62]	Cross-sectional	62 2.6 49.2 (43.7–54.5) **	RRMS: 39 SPMS: 23	13.7 (10.1–20) **	3 (2–4) **	NR	-	-	NR	Serum ELISA	3T Phillips	sGFAP is a biomarker for MS pathology-related astrocytopathy and related diffuse white matter damage.	7
M. Niiranen Finland 2021 [63]	Case- control	63 2.7 50.3 (21–78) ^β^	RRMS: 63	16.6 (3–43) ^β^	2.2 (1–3) ^β^	NR	-	-	14 1 47.4 (31–63) ^β^	Serum ELISA	NR	sGFAP measurement cannot separate RRMS patients with and without treatment after a long history of the disease.	7
C. Liu China 2021 [64]	Case- control	98 2 31 (27–38) **	NR	5 (2–9) **	2 (1.5–3) **	102 8.3 39.5 (29.2–53) **	5 (2.6–9) **	3 (2–3.5) **	84 1.8 28 (26–34) **	Serum ELISA	NR	sGFAP and sNfL are potential blood biomarkers for diagnosing and monitoring NMOSD and MS.	9
J. Giarraputo USA 2021 [65]	Cohort	25 2.6 62 (53–67) **	PPMS: 25	NR	NR	NR	-	-	NR	Serum ELISA	NR	Results suggest a limited role for GFAP in primary progressive disease management.	5
K. Edwards USA 2021 [72]	Case- control	16 7 55.4 (8.9)	SPMS: 16	NR	4.3 (1.5)	NR	-	-	4 1 39 (11)	Serum CSF ELISA	3T Siemens	GFAP level showed a correlation to disease activity in pwSPMS.	5
X. Chang China 2021 [66]	Case- control	31 1.2 31 (25–38) **	RRMS: 31	17 (5–76) **	2 (1.5–3) **	51 6.3 37 (24–48) **	17 (5–66) **	3 (1.5–4) **	28 1.3 35 (24–47) **	Serum ELISA	NR	sGFAP level is associated with disease severity in NMOSD patients.	7
O. Aktas Germany 2021 [19]	Case- control	NR	-	-	-	215 9.2 43.1 (11.8)	2.5 (3.3)	3.9 (1.8)	85 9.6 43.4 (12.9)	Serum ELISA	NR	sGFAP may serve as a biomarker of NMOSD activity, attack risk, and treatment effects.	9
A. Huss Germany 2020 [73]	Case-control	86 1.4 42.9 (27–59) ^β^	PMS: 39 RRMS: 47	NR	NR	NR	-	-	NR	Serum CSF ELISA	NR	GFAP mechanisms in differentiating between PMS and RMS in the CSF and monitoring disease progression are useful.	7
H. Kim South Korea 2020 [67]	Cross-sectional	NR	-	-	-	33 10 51 (43–59) **	4 (1.5–8) **	3 (2–4.2) **	NR	Serum ELISA	NR	NfL and GFAP are considered to represent neuroaxonal and astrocyte damage	6
I. Kleerekooper UK 2020 [15]	Case-control	69 3.1 42.1 (10.6)	NR	-	-	39 2.8 45.2 (16.8)	NR	NR	37 1 43.2 (11.1)	CSF ELISA	NR	Elevated GFAP level identify NMOSD patients suitable to undergo in-depth autoimmune screening for astrocytic antibodies.	7
E. Lee South Korea 2020 [68]	Cohort	117 2.7 45 (34–54) **	NR	-	2 (1–4) **	63 9.5 54 (46–60) **	NR	3.5 (2–5) **	NR	Serum ELISA	NR	sGFAP level reflects disease severity and varies significantly with NMOSD patients.	8
I. Sharquie Iraq 2020 [69]	Case- control	NR	-	-	-	24 2 30.2 (6.9)	NR	NR	24 1.8 31.7 (5.5)	Serum ELISA	NR	Measuring sGFAP in NMOSD is helpful in the diagnosis of the condition.	5
X. Ayrignac France 2020 [70]	Cross-sectional	129 3 41.5 (11)	RRMS: 111 PPMS: 18	6.7 (7.1)	1.7 (0–3) **	NR	-	-	NR	Serum ELISA	3T Skyra Siemens	s-GFAP was correlated with white matter lesion load and inversely correlated with white and grey matter volume.	7
E. Oguz Turkey 2019 [71]	Case- control	51 0.3 36.4 (9.8)	CIS: 4 RRMS: 36 SPMS: 8 PPMS: 3	NR	5.2 (1.9)	NR	-	-	37 0.48 40.4 (12.4)	Serum ELISA	MRI	There was no difference between patient and control groups in terms of GFAP level.	7
T. Kalatha Greece 2019 [74]	Case-control	87 3.2 41.1 (12)	RRMS: 56 SPMS: 8 PPMS: 4 CIS: 19	7.2 (8.8)	2.6 (1.7)	NR	-	-	21 0.7 44.2 (12.8)	Serum CSF ELISA	NR	Biomarkers may help evaluate neuronal damage in active MS and reflect secondary pathogenetic mechanisms of repair or progression.	6
A. Abdelhak Germany 2019 [75]	Cross-sectional	93 1.1 49 (44–57) **	PPMS: 93	4.5 (2–12) **	4.5 (3.5–6.5) **	NR	-	-	NR	Serum CSF ELISA	NR	Results highlight a particular role of the astrocytes in PPMS and mark the potential of GFAP as a disease severity marker.	7
L. Novakova Sweden 2018 [35]	Case-control	159 2.3 37.4 (18–67) ^β^	RRMS: 136 PMS: 51	4.2 (0–39) ^β^	2.2 (1)	NR	-	-	51 0.9 27 (20–49) ^β^	CSF ELISA	3T NR	GFAP level had diagnostic value, and these biomarkers could be included in diagnostic work-ups for multiple sclerosis.	8
A. Abdelhak Germany 2018 [14]	Case-control	80 NR 43.2 (13.3)	RRMS: 42 SPMS: 13 PPMS: 25	8.7 (21.5)	3.7 (1.9)	NR	-	-	20 NR 40.7 (19.9)	Serum CSF ELISA	1.5T Siemens	GFAP might indicate a possible role of astrocytes in the neuroaxonal demise of MS.	7
L. Novakova Sweden 2017 [36]	Case-control	59 1.5 37 (17–59) ^β^	RRMS: 59	8.4 (0–23) ^β^	2.5 (0–7.5) *	NR	-	-	39 0.5 34 (21–56) ^β^	CSF ELISA	3T NR	The results indicate that the CSF level of GFAP correlates with the clinical and radiological disease activity.	6
R. Kassubek Germany 2017 [11]	Case-control	18 1.5 26 (23–29) **	RRMS: 18	NR	NR	NR	-	-	35 3.3 43 (30–52) **	CSF ELISA	1.5T Siemens	GFAP seems to be a useful biomarker for highly active acute inflammation in patients with RRMS.	5
I. Hakansson Sweden 2017 [37]	Case-control	41 3.5 30.2 (9.2)	RRMS: 22 CIS: 19	0.6 (0.9)	2 (1)	NR	-	-	22 3.4 32 (26–41) **	CSF ELISA	1.5T Philips	The study demonstrates the potential prognostic value of GFAP in baseline CSF in RRMS.	6
J. Burman Sweden 2014 [38]	Case-control	64 1.6 43.9 (9.6)	RRMS: 44 SPMS: 20	13.2 (8.3)	3.2 (1.3)	NR	-	-	15 2 40 (15)	CSF ELISA	1.5T NR	GFAP provides a direct means to measure tissue damage and is a useful addition to our methods for evaluating MS.	7
M. Axelsson Sweden 2013 [39]	Case control	35 0.7 48 (22–65) ^β^	SPMS: 30 PPMS: 5	15 (2–29) *	6 (3–8) *	NR	-	-	14 0.5 42 (31–61) ^β^	CSF ELISA	3T NR	The determination of GFAP levels in CSF is a potential surrogate marker for treatment efficacy.	7
R. Madeddu Italy 2013 [40]	Cross-sectional	33 2.3 39.3 (13.2)	RRMS: 24 SPMS: 7 PPMS: 1	NR	NR	NR	-	-	NR	CSF ELISA	NR	Higher levels of b-Tub II and GFAP were found in remitting MS forms.	5
M. Storoni UK 2012 [20]	Cross-sectional	47 2.8 41 (21–66) *	RRMS: 47	NR	NR	77 6.4 41 (14–66) *	NR	NR	NR	Serum ELISA	NR	Serum GFAP levels were not a diagnostic value for the laboratory differential diagnosis of NMO.	8
M. Gunnarsson Sweden 2011 [41]	Case-control	92 1.4 37.3 (14–59) ^β^	RRMS: 92	9.6 (0.5–28) ^β^	3.8 (2.3)	NR	-	-	28 0.4 43 (27–62) ^β^	CSF ELISA	NR	GFAP anticipated that highly effective anti-inflammatory treatment can reduce axonal loss.	7
M. Axelsson Sweden 2011 [42]	Case-control	25 0.5 41 (21–59) ^β^	RRMS: 15 SPMS: 10	11 (1–40) ^β^	3.9 (2.2)	NR	-	-	28 2.5 33 (18–53) ^β^	CSF ELISA	NR	GFAP is a potential biomarker for MS progression and may have a role in clinical trials for assessing the impact of therapies on MS progression.	7
R. Takano Japan 2010 [76]	Cross-sectional	27 4.4 34.9 (11.7)	NR	5.2 (4.6)	3.8 (1.7)	33 33:0 43.8 (13.4)	6.2 (5.2)	5.4 (1.9)	NR	Serum CSF ELISA	NR	Astrocytic damage reflected by elevated GFAP is clinically relevant.	8
T. Misu Japan 2009 [43]	Cross-sectional	10 1 31 (26–51) *	NR	4.9 (2.2–13) *	3 (2–8) *	10 10:0 42 (33–59) *	3.3 (0–14.3) *	6.3 (3–8.5) *	NR	CSF ELISA	NR	CSF-GFAP may be a clinically useful biomarker in NMO, and astrocytic damage is strongly suggested in the acute phase of NMO.	5
N. Norgren Sweden 2004 [44]	Case-control	99 1.8 38 (29.5–44) **	RRMS: 58 SPMS: 21 PPMS: 15 PRMS: 5	5 (3–8) **	2 (1.5–3.5) **	NR	-	-	25 2.1 35 (28–44.5) **	CSF ELISA	NR	CSF level of GFAP may have prognostic value in multiple sclerosis.	8
S. Haghighi Sweden 2004 [45]	Case-control	47 NR 44	NR	NR	NR	NR	-	-	50 NR 33	CSF ELISA	NR	Our main finding was the normal CSF concentration of GFAP in the MS individuals.	5
C. Malmestrom Sweden 2003 [46]	Case-control	66 1.64 39.6 (8.2)	RRMS: 41 SPMS: 25	14.9 (5.6)	4.1 (1.1)	NR	-	-	50 0.4 36.2 (8.4)	CSF ELISA	NR	GFAP may serve as a biomarker for disease progression, probably reflecting the increasing rate of astrogliosis.	7
A. Petzold UK 2002 [18]	Case-control	51 0.8 46 (8.4)	RRMS: 20 SPMS: 21 PPMS: 10	20.4 (7.9)	3.5 (0–8) *	NR	-	-	51 0.4 41.6 (7.9)	CSF ELISA	NR	GFAP correlated with disability scale and may, therefore, be a marker for irreversible damage.	6

* Median (Range), ** Median (IQR), ^β^ Mean (Range). CIS: Clinically Isolated Syndrome, CSF: Cerebrospinal Fluid, ELISA: Enzyme-linked Immunosorbent Assay, GFAP: Glial Fibrillary Acidic Protein, MRI: Magnetic Resonance Imaging, MS: Multiple Sclerosis, NfL: Neurofilament Light, NMOSD: Neuromyelitis Optica Spectrum Disorder, NR: Not Reported, PMS: Progressive Multiple Sclerosis, PPMS: Primary Progressive Multiple Sclerosis, PwMS: People with Multiple Sclerosis, PwNMOSD: People with Neuromyelitis Optica Spectrum Disorder, RIS: Radiologically Isolated Syndrome, SPMS: Secondary Progressive Multiple Sclerosis.

**Table 2:** The results of a meta-analysis of pooled standard mean difference of GFAP between MS, NMOSD, and HCs.

Participants	Sample	Model	N. of Studies	Pooled SMD	95% CI	*p*-Value	I^2^	*P*-Heterogeneity	Publication Bias			
									Begg		Egger	
									Score	*p*-Value	Bias	*p*-Value
MS vs. HCs	CSF	Random	13	0.7	0.54 to 0.86	**<0.001**	29%	0.16	−2	0.9	0.8	0.7
	Serum	Random	8	0.54	0.1 to 0.99	**0.02**	90%	**<0.01**	4	0.62	−0.59	0.86
PMS vs. RRMS	CSF	Random	7	0.45	0.22 to 0.69	**<0.001**	34%	0.17	−9	0.17	−3.2	0.25
	Serum	Random	6	0.5	0.25 to 0.75	**<0.001**	53%	0.06	−1	0.85	0.7	0.77
NMOSD vs. HCs	Serum	Random	7	0.9	0.73 to 1.07	**<0.001**	10%	0.35	−7	0.29	−2.1	0.17

Significant *p*-values are presented in bold. CSF: Cerebrospinal Fluid, HCs: Healthy Controls, GFAP: Glial Fibrillary Acidic Protein, N: Number, NMOSD: Neuromyelitis Optica Spectrum Disorder, PMS: Progressive Multiple Sclerosis, RRMS: Relapsing–remitting multiple sclerosis, SMD: Standard Mean Difference.

**Table 3:** The results of a meta-analysis of pooled correlation coefficients of GFAP with demographic and clinical characteristics in MS and NMOSD.

Characteristics	Disorder	Sample	Model	N. of Studies	N. of Patients	Pooled Correlation Coefficients	95% CI	*p*-Value	I^2^	*P*-Heterogeneity	Publication Bias			
											Begg		Egger	
											Score	*p*-Value	Bias	*p*-Value
Disease Duration	MS	Serum	Random	5	403	0.28	0.15 to 0.41	**<0.001**	53%	0.07	−4	0.33	−11	0.26
EDSS	MS	CSF	Random	11	676	0.43	0.26 to 0.59	**<0.001**	91%	**<0.01**	−19	0.14	−8.7	**0.017**
		Serum	Random	7	687	0.36	0.23 to 0.49	**<0.001**	78%	**<0.01**	−13	0.051	−7.6	**0.011**
	NMOSD	Serum	Random	4	326	0.35	0.26 to 0.45	**<0.001**	0%	0.7	2	0.5	0.58	0.57
Nfl	MS	CSF	Random	6	495	0.39	0.29 to 0.49	**<0.001**	38%	0.15	−3	0.57	−1.6	0.45
		Serum	Random	8	968	0.42	0.32 to 0.52	**<0.001**	76%	**<0.01**	−6	0.45	−4.6	0.06
T2LV	MS	CSF + Serum	Random	3	410	0.37	0.29 to 0.46	**<0.001**	0%	0.57	−1	0.6	−1.7	0.52

Significant *p*-values are presented in bold. CSF: Cerebrospinal Fluid, GFAP: Glial Fibrillary Acidic Protein, HCs: Healthy Controls, N: Number, NMOSD: Neuromyelitis Optica Spectrum Disorder, PMS: Progressive Multiple Sclerosis, RRMS: Relapsing–remitting multiple sclerosis.

## Supplementary Materials

The following supporting information can be downloaded at: https://www.mdpi.com/article/10.3390/medicina60071050/s1, Supplementary S1: The syntax that was used in searching each database. Supplementary S2: The Forest plots, Funnel plots, and sensitivity analyses of pooled standard mean difference and correlations of glial fibrillary acidic protein (GFAP) levels in people with multiple sclerosis, neuromyelitis optica spectrum disorder and healthy controls.
