# Supplementary material for: The Role of Glial Fibrillary Acidic Protein as a Biomarker in Multiple Sclerosis and Neuromyelitis Optica Spectrum Disorder: A Systematic Review and Meta-Analysis
Source: Medicina (Kaunas). 2024 Jun 26;60(7):1050. doi: 10.3390/medicina60071050 (PMC11279275; doi:10.3390/medicina60071050)
Supplement: Supplementary file 1 [file medicina-60-01050-s001.zip › Supplementary S1.pdf]

Supplementary S1. syntax used in each database.

N	All Databases	Results 4109
	PUBMED/MEDLINE	
1.	(“Neuromyelitis Optica” OR "NMO Spectrum Disorder" OR "NMO Spectrum Disorders" OR "Neuromyelitis Optica (NMO) Spectrum Disorder" OR "Neuromyelitis Optica Spectrum Disorders" OR "Devic Neuromyelitis Optica" OR "Devic Neuromyelitis Opticas" OR ("Neuromyelitis Optica” AND Devic) OR ("Neuromyelitis Opticas” AND Devic) OR "Devic's Disease" OR "Devics Disease" OR "Disease, Devic's" OR "Devic Disease" OR (Disease AND Devic) OR ("Devic Syndrome") OR (Syndrome AND Devic) OR "Devic's Syndrome" OR "Devics Syndrome" OR "Syndrome, Devic's" OR "Devic's Neuromyelitis Optica" OR "Devics Neuromyelitis Optica" OR ("Neuromyelitis Optica” AND Devic's) OR "Neuromyelitis Optica Spectrum Disorder" OR "Neuromyelitis Optica (NMO) Spectrum Disorders")<br>AND<br><br>(“Sclerosis, Multiple” OR “Sclerosis, Disseminated” OR “Disseminated Sclerosis” OR “MS (Multiple Sclerosis)” OR “Multiple Sclerosis, Acute Fulminating”)<br><br>AND<br><br>(“Glial Intermediate Filament Protein” OR “Glial Fibrillary Acid Protein” OR “Astroprotein” OR “GFA-Protein” OR “GFA Protein”)	780
	EMBASE	
2.	(‘Sclerosis, Multiple’ OR ‘Sclerosis, Disseminated’ OR ‘Disseminated Sclerosis’ OR ‘MS (Multiple Sclerosis)’ OR ‘Multiple Sclerosis, Acute Fulminating’)<br><br>AND<br><br>('neuromyelitis optica'/exp OR 'neuromyelitis optica' OR 'nmo spectrum disorder' OR 'nmo spectrum disorders' OR 'neuromyelitis optica (nmo) spectrum disorder' OR 'neuromyelitis optica spectrum disorders' OR 'devic neuromyelitis optica' OR 'devic neuromyelitis opticas' OR (('neuromyelitis optica'/exp OR 'neuromyelitis optica') AND devic) OR ('neuromyelitis opticas' AND devic) OR 'devics disease' OR 'disease, devics' OR 'devic disease'/exp OR 'devic disease' OR (('disease'/exp OR disease) AND devic) OR 'devic syndrome'/exp OR 'devic syndrome' OR (('syndrome'/exp OR syndrome) AND devic) OR 'devics syndrome' OR 'syndrome, devics' OR 'devics neuromyelitis optica' OR (('neuromyelitis optica'/exp OR 'neuromyelitis optica') AND devics) OR 'neuromyelitis optica spectrum disorder'/exp OR 'neuromyelitis optica spectrum disorder' OR 'neuromyelitis optica (nmo) spectrum disorders')	1918

	AND<br><br>(‘Glial Intermediate Filament Protein’ OR ‘Glial Fibrillary Acid Protein’ OR ‘Astroprotein’ OR ‘GFA-Protein’ OR ‘GFA Protein’)	
	SCOPUS	
3.	TITLE-ABS((“Sclerosis, Multiple” OR “Sclerosis, Disseminated” OR “Disseminated Sclerosis” OR “MS (Multiple Sclerosis)” OR “Multiple Sclerosis, Acute Fulminating”)<br><br>AND<br><br>(“Neuromyelitis Optica” OR "NMO Spectrum Disorder" OR "NMO Spectrum Disorders" OR "Neuromyelitis Optica (NMO) Spectrum Disorder" OR "Neuromyelitis Optica Spectrum Disorders" OR "Devic Neuromyelitis Optica" OR "Devic Neuromyelitis Opticas" OR ("Neuromyelitis Optica” AND Devic) OR ("Neuromyelitis Opticas” AND Devic) OR "Devic's Disease" OR "Devics Disease" OR "Disease, Devic's" OR "Devic Disease" OR (Disease AND Devic) OR ("Devic Syndrome") OR (Syndrome AND Devic) OR "Devic's Syndrome" OR "Devics Syndrome" OR "Syndrome, Devic's" OR "Devic's Neuromyelitis Optica" OR "Devics Neuromyelitis Optica" OR ("Neuromyelitis Optica” AND Devic's) OR "Neuromyelitis Optica Spectrum Disorder" OR "Neuromyelitis Optica (NMO) Spectrum Disorders")<br><br>AND<br><br>(“Glial Intermediate Filament Protein” OR “Glial Fibrillary Acid Protein” OR “Astroprotein” OR “GFA-Protein” OR “GFA Protein”))	659
	Web of Sciences	

4.	TS=((“Sclerosis, Multiple” OR “Sclerosis, Disseminated” OR “Disseminated Sclerosis” OR “MS (Multiple Sclerosis)” OR “Multiple Sclerosis, Acute Fulminating”)) and Preprint Citation Index (Exclude – Database)<br><br>AND<br><br>S=((“Neuromyelitis Optica” OR "NMO Spectrum Disorder" OR "NMO Spectrum Disorders" OR "Neuromyelitis Optica (NMO) Spectrum Disorder" OR "Neuromyelitis Optica Spectrum Disorders" OR "Devic Neuromyelitis Optica" OR "Devic Neuromyelitis Opticas" OR ("Neuromyelitis Optica” AND Devic) OR ("Neuromyelitis Opticas” AND Devic) OR "Devic's Disease" OR "Devics Disease" OR "Disease, Devic's" OR "Devic Disease" OR (Disease AND Devic) OR ("Devic Syndrome") OR (Syndrome AND Devic) OR "Devic's Syndrome" OR "Devics Syndrome" OR "Syndrome, Devic's" OR "Devic's Neuromyelitis Optica" OR "Devics Neuromyelitis Optica" OR ("Neuromyelitis Optica” AND Devic's) OR "Neuromyelitis Optica Spectrum Disorder" OR "Neuromyelitis Optica (NMO) Spectrum Disorders")) and Preprint Citation Index (Exclude – Database)<br><br>AND<br><br>S=((“Glial Intermediate Filament Protein” OR “Glial Fibrillary Acid Protein” OR “Astroprotein” OR “GFA-Protein” OR “GFA Protein”)) and Preprint Citation Index (Exclude – Database)	752
