# Supplementary material for: The Role of Glial Fibrillary Acidic Protein as a Biomarker in Multiple Sclerosis and Neuromyelitis Optica Spectrum Disorder: A Systematic Review and Meta-Analysis
Source: Medicina (Kaunas). 2024 Jun 26;60(7):1050. doi: 10.3390/medicina60071050 (PMC11279275; doi:10.3390/medicina60071050)
Supplement: Supplementary file 1 [file medicina-60-01050-s001.zip › Supplementary S2.pdf]

Supplementary S2. Forest plots, Funnel plots, and sensitivity analyses of pooled standard mean difference (SMD) and correlations of glial fibrillary acidic protein (GFAP) levels in multiple sclerosis (MS), neuromyelitis optica spectrum disorder (NMOSD) and healthy controls (HCs).

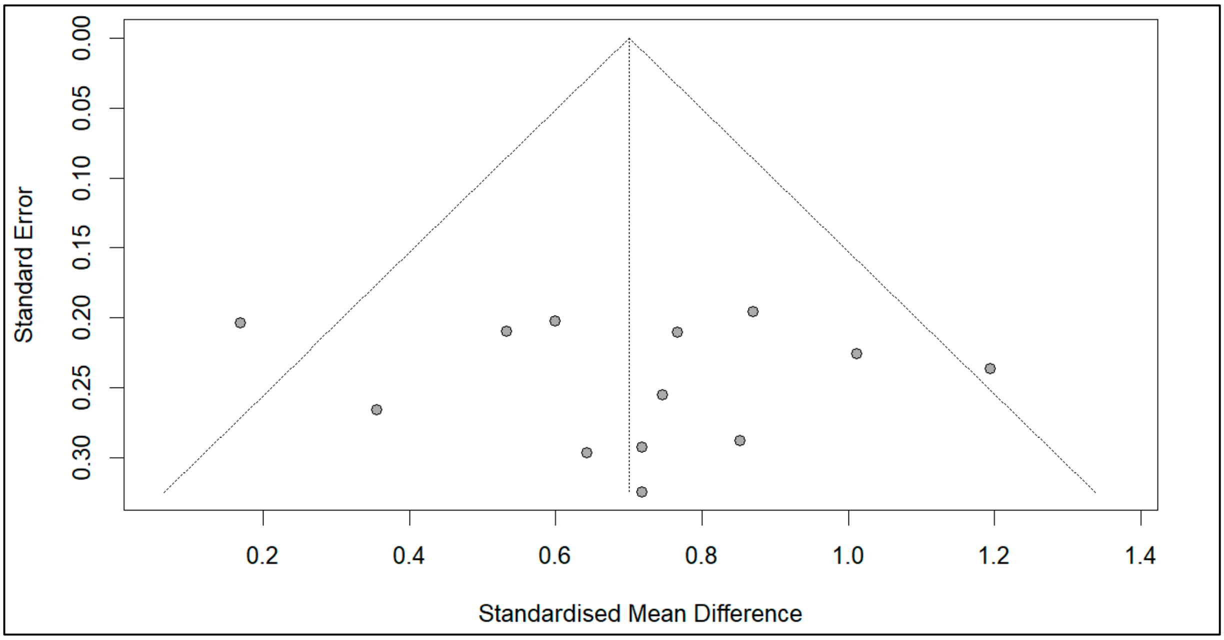

Figure S1. Funnel plot of the meta-analysis of pooled SMD of cerebrospinal fluid level of GFAP (cGFAP) between PwMS and HCs.

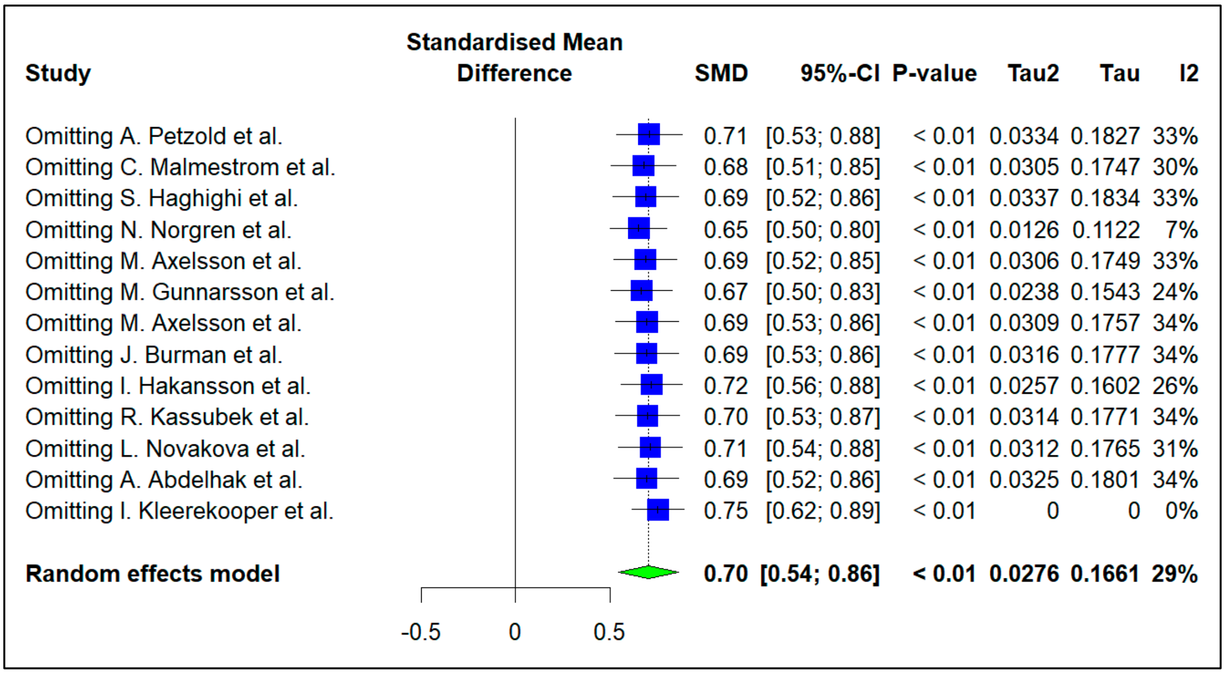

Figure S2. Sensitivity analysis of the meta-analysis of pooled SMD of cGFAP between PwMS and HCs.

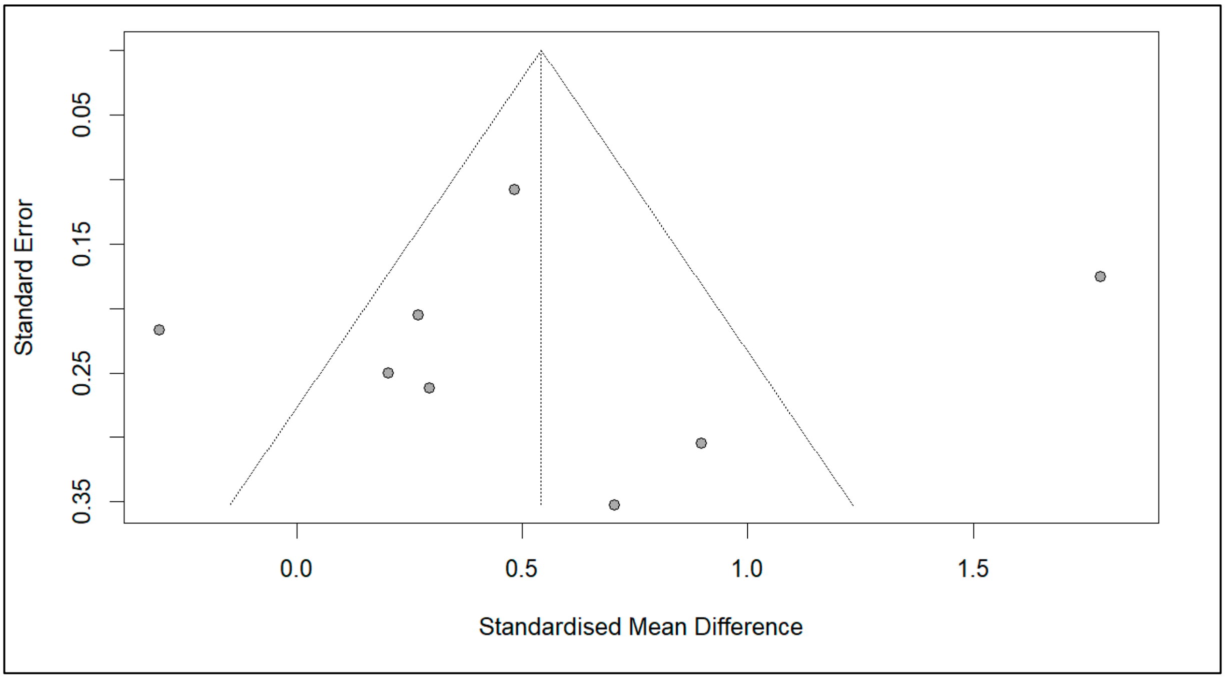

Figure S3. Funnel plot of the meta-analysis of pooled SMD of serum level of GFAP (sGFAP) between PwMS and HCs.

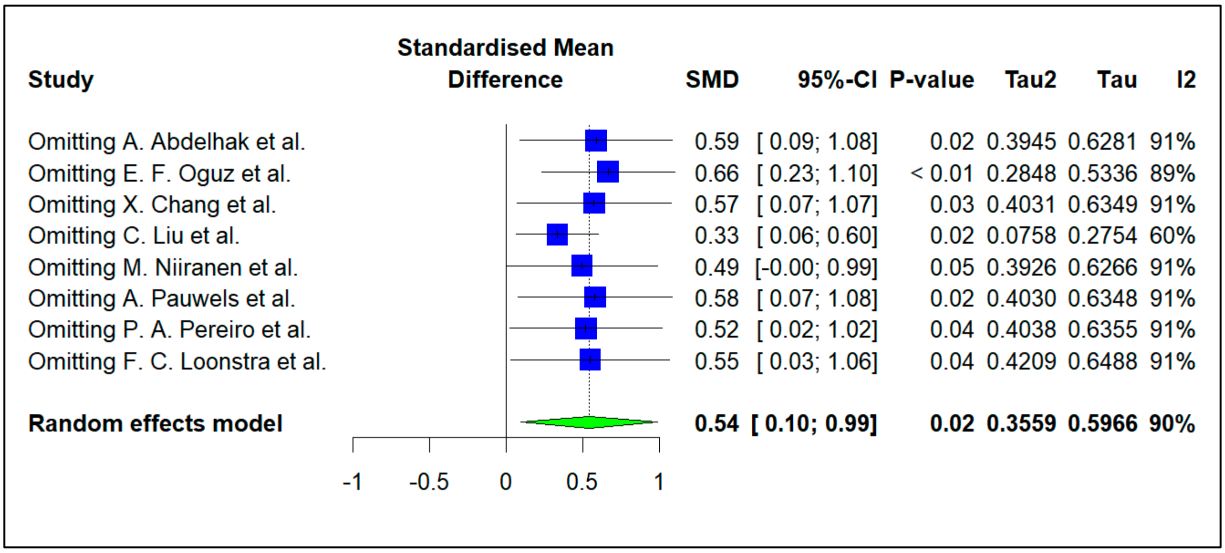

Figure S4. Sensitivity analysis of the meta-analysis of pooled SMD of sGFAP between PwMS and HCs.

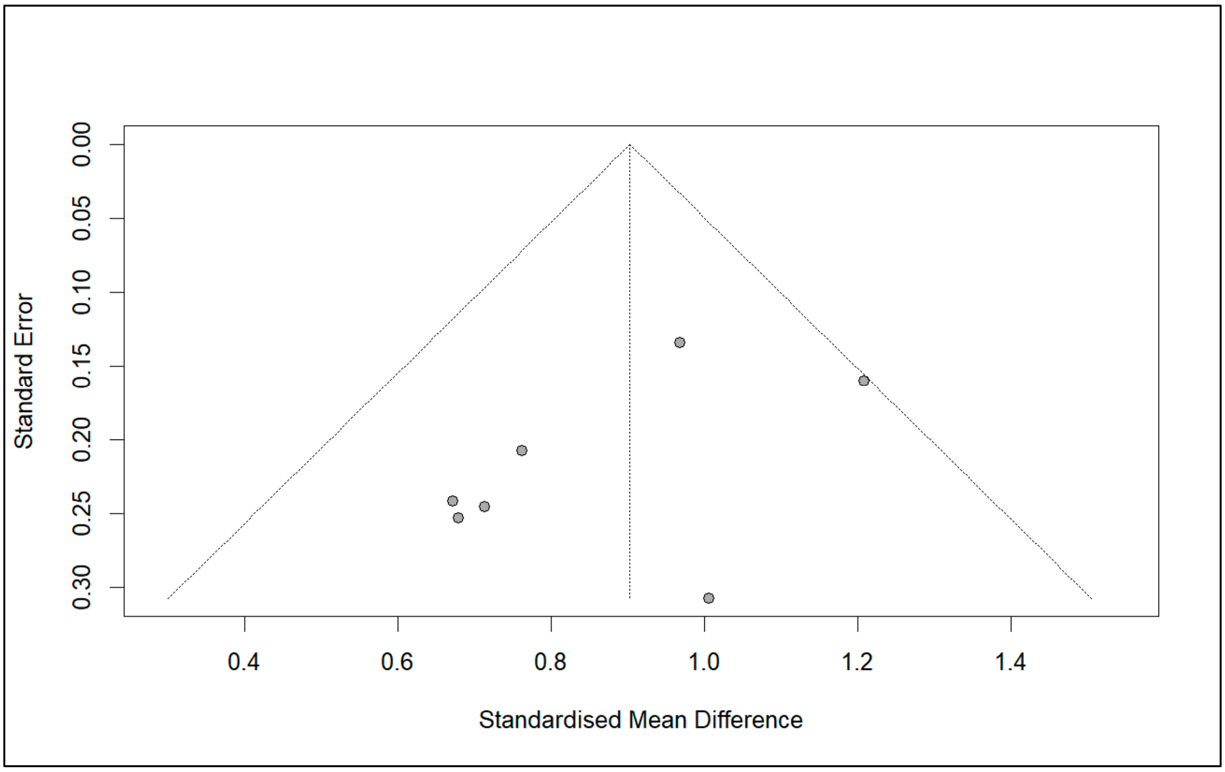

Figure S5. Funnel plot of the meta-analysis of pooled SMD of sGFAP between PwNMOSD and HCs.

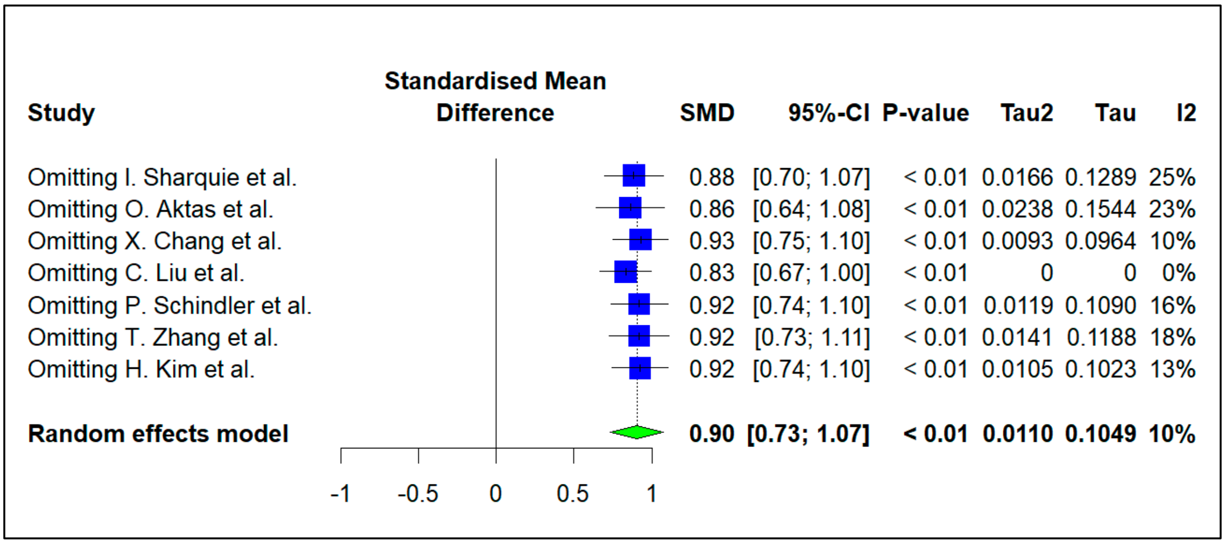

Figure S6. Sensitivity analysis of the meta-analysis of pooled SMD of sGFAP between PwNMOSD and HCs.

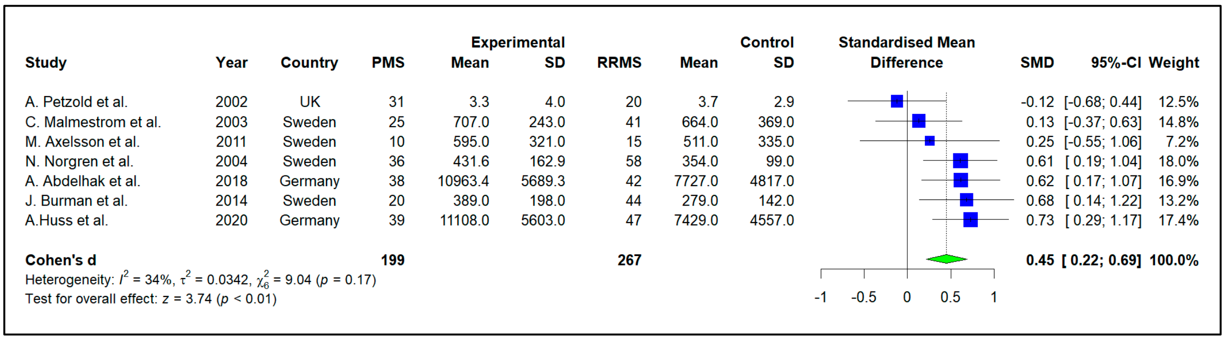

Study	Year	Country	PMS	Experimental Mean	Experimental SD	RRMS	Control Mean	Control SD	SMD	95%-CI	Weight
A. Petzold et al.	2002	UK	31	3.3	4.0	20	3.7	2.9	-0.12	[-0.68; 0.44]	12.5%
C. Malmestrom et al.	2003	Sweden	25	707.0	243.0	41	664.0	369.0	0.13	[-0.37; 0.63]	14.8%
M. Axelsson et al.	2011	Sweden	10	595.0	321.0	15	511.0	335.0	0.25	[-0.55; 1.06]	7.2%
N. Norgren et al.	2004	Sweden	36	431.6	162.9	58	354.0	99.0	0.61	[0.19; 1.04]	18.0%
A. Abdelhak et al.	2018	Germany	38	10963.4	5689.3	42	7727.0	4817.0	0.62	[0.17; 1.07]	16.9%
J. Burman et al.	2014	Sweden	20	389.0	198.0	44	279.0	142.0	0.68	[0.14; 1.22]	13.2%
A.Huss et al.	2020	Germany	39	11108.0	5603.0	47	7429.0	4557.0	0.73	[0.29; 1.17]	17.4%
**Cohen's d**			**199**			**267**			**0.45**	**[0.22; 0.69]**	**100.0%**

Heterogeneity: I^2 = 34%, τ^2 = 0.0342, χ^2_6 = 9.04 (p = 0.17)
Test for overall effect: z = 3.74 (p < 0.01)

Figure S7. Forest plot of the meta-analysis of pooled SMD of cGFAP progressive MS (PMS) patients and relapsing-remitting MS (RRMS) patients.

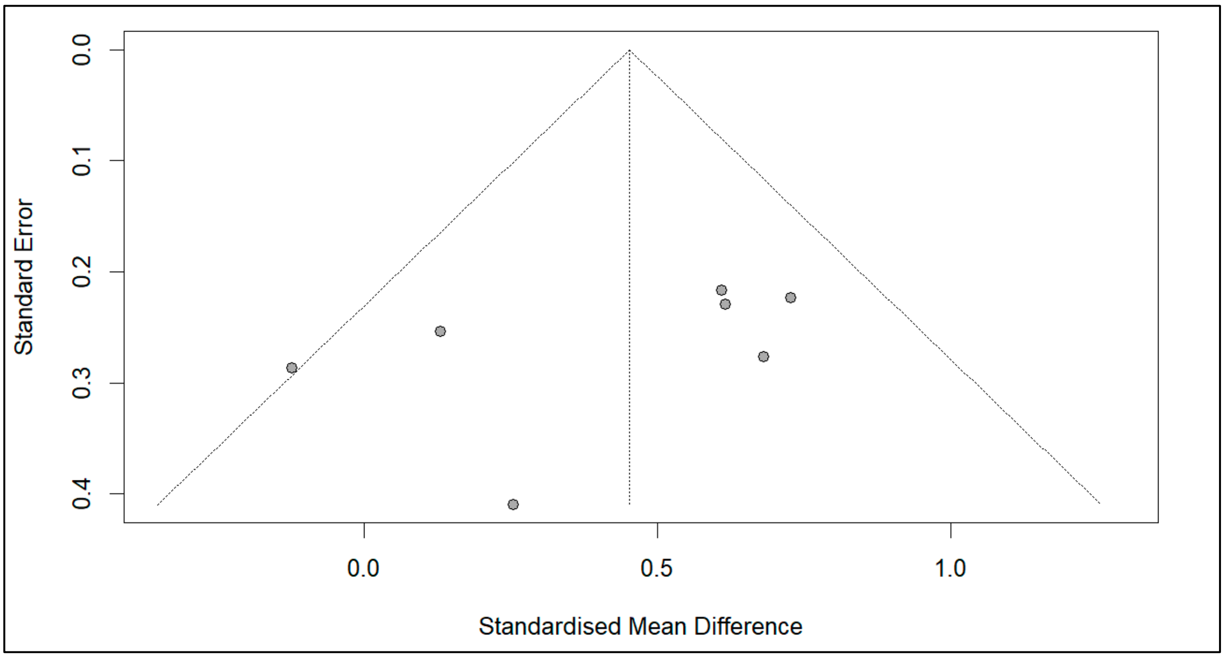

Figure S8. Funnel plot of the meta-analysis of pooled SMD of cGFAP between PMS and RRMS.

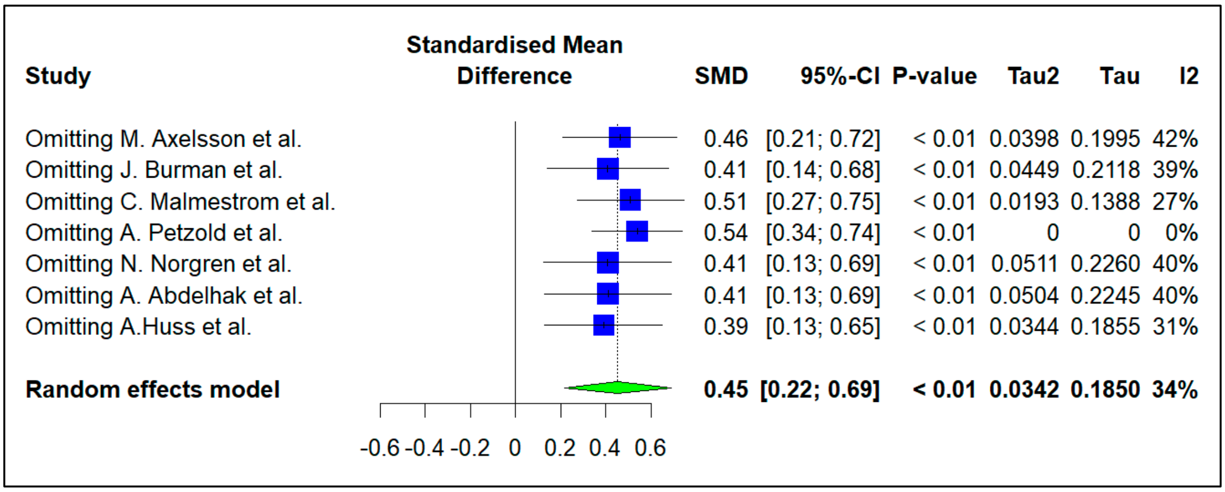

Figure S9. Sensitivity analysis of the meta-analysis of pooled SMD of cGFAP between PMS and RRMS.

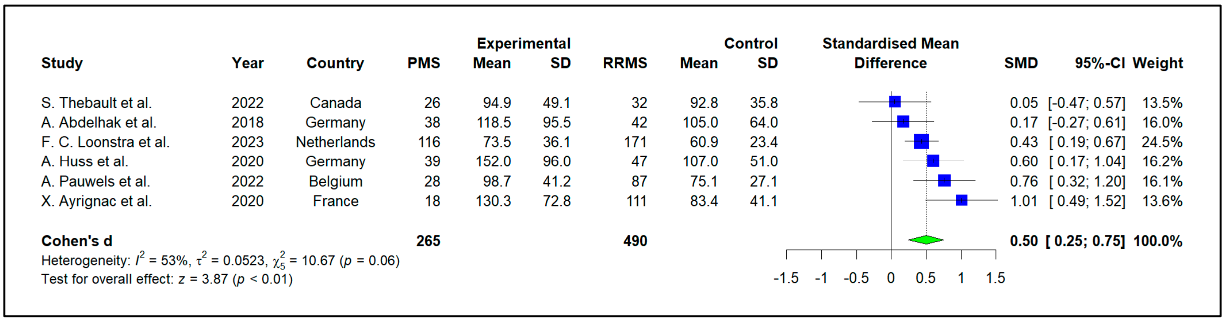

Figure S10. Forest plot of the meta-analysis of pooled SMD of sGFAP between PMS and RRMS.

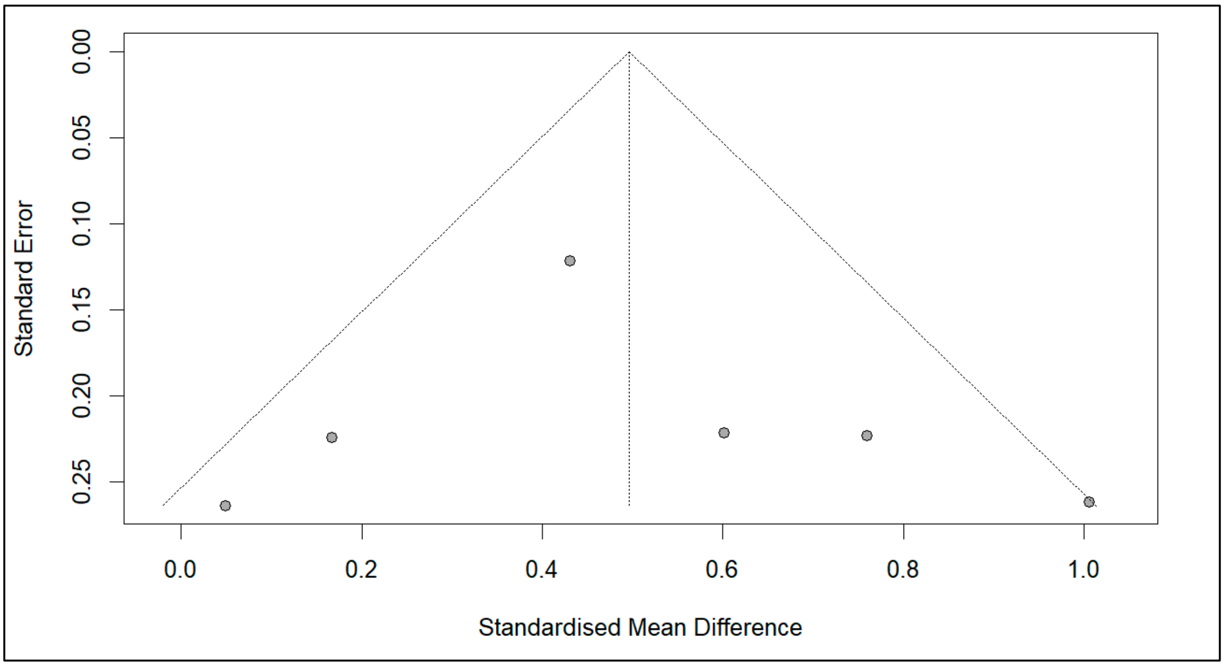

Figure S11. Funnel plot of the meta-analysis of pooled SMD of sGFAP between PMS and RRMS.

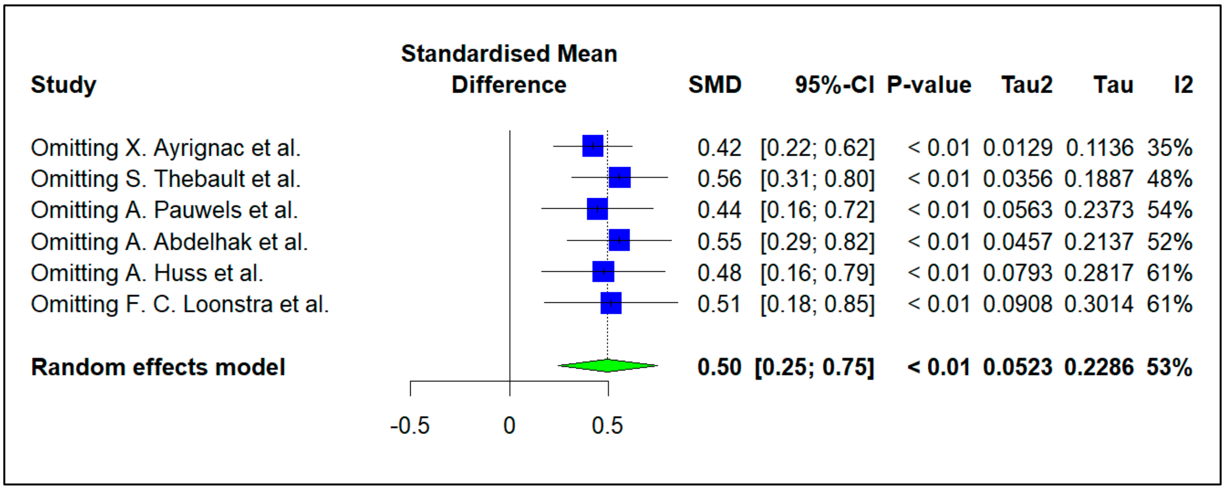

Figure S12. Sensitivity analysis of the meta-analysis of pooled SMD of sGFAP between PMS and RRMS.

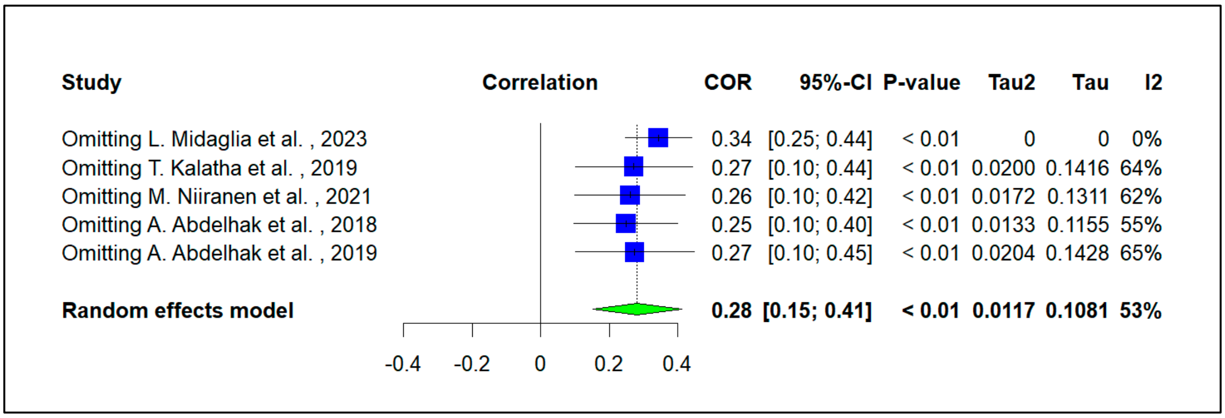

Figure S13. Sensitivity analysis of the meta-analysis of the correlation between sGFAP and disease duration in PwMS.

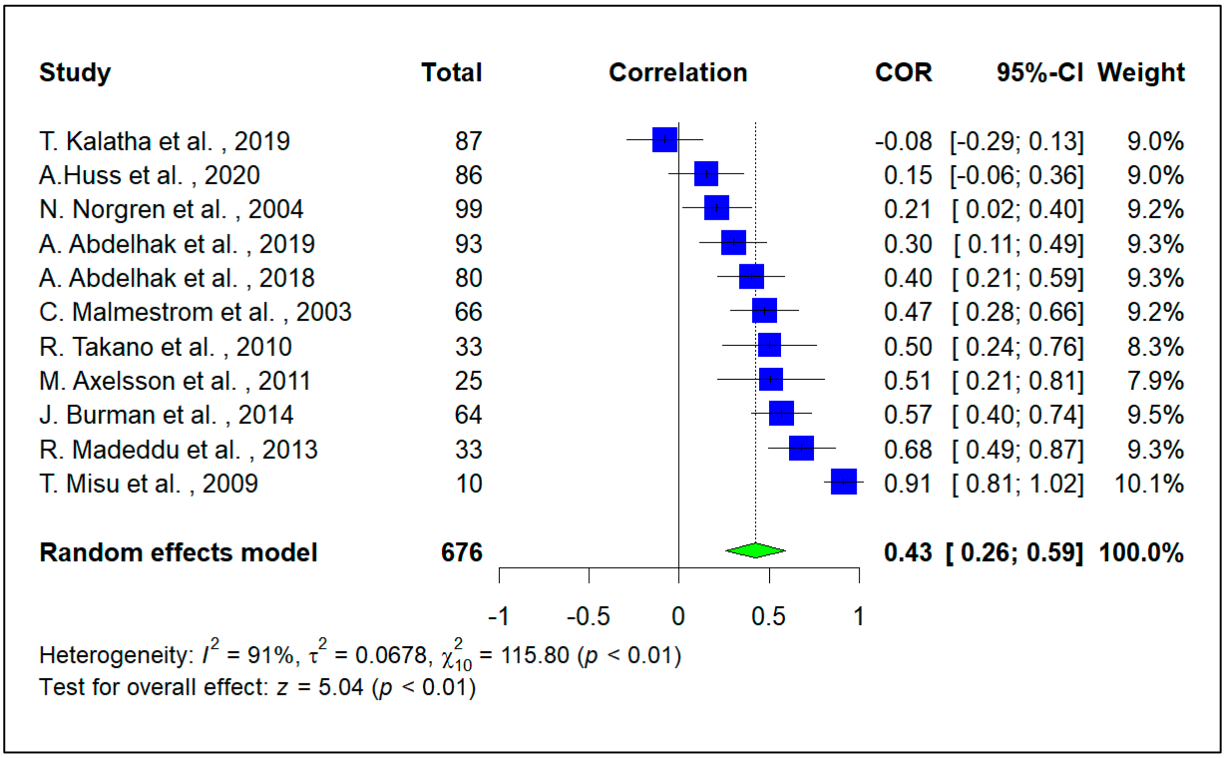

Figure S14. Forest plot of the meta-analysis of the correlation between cGFAP and expanded disability status scale (EDSS) in PwMS.

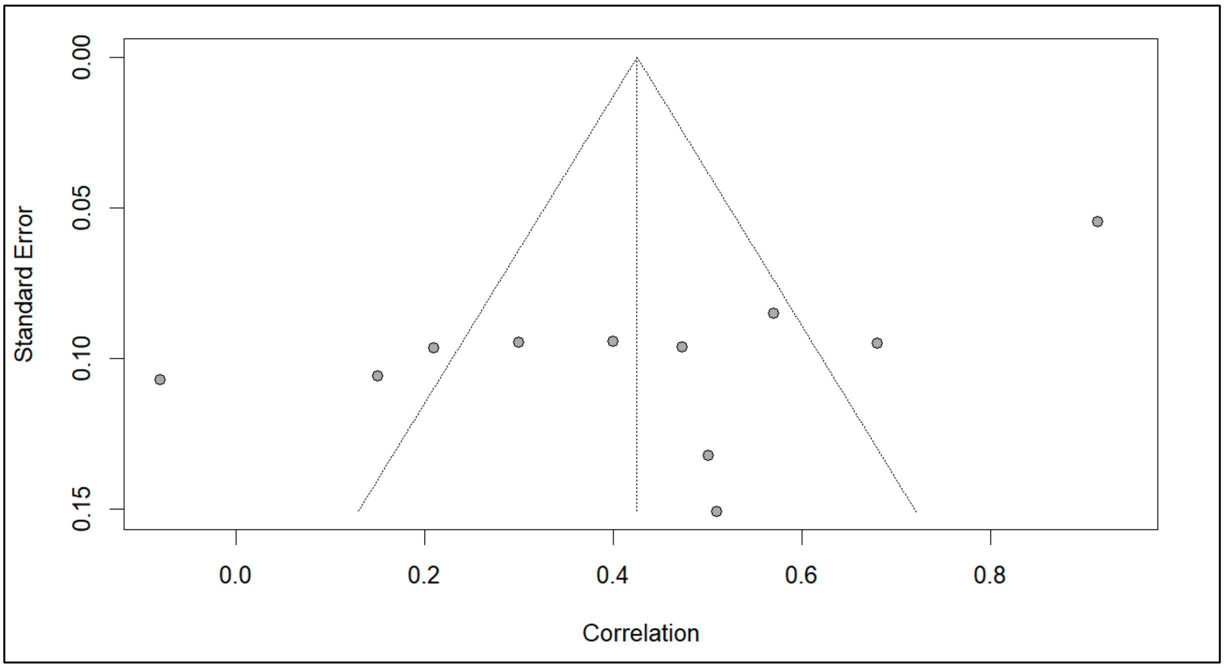

Figure S15. Funnel plot of the meta-analysis of the correlation between cGFAP and EDSS in PwMS.

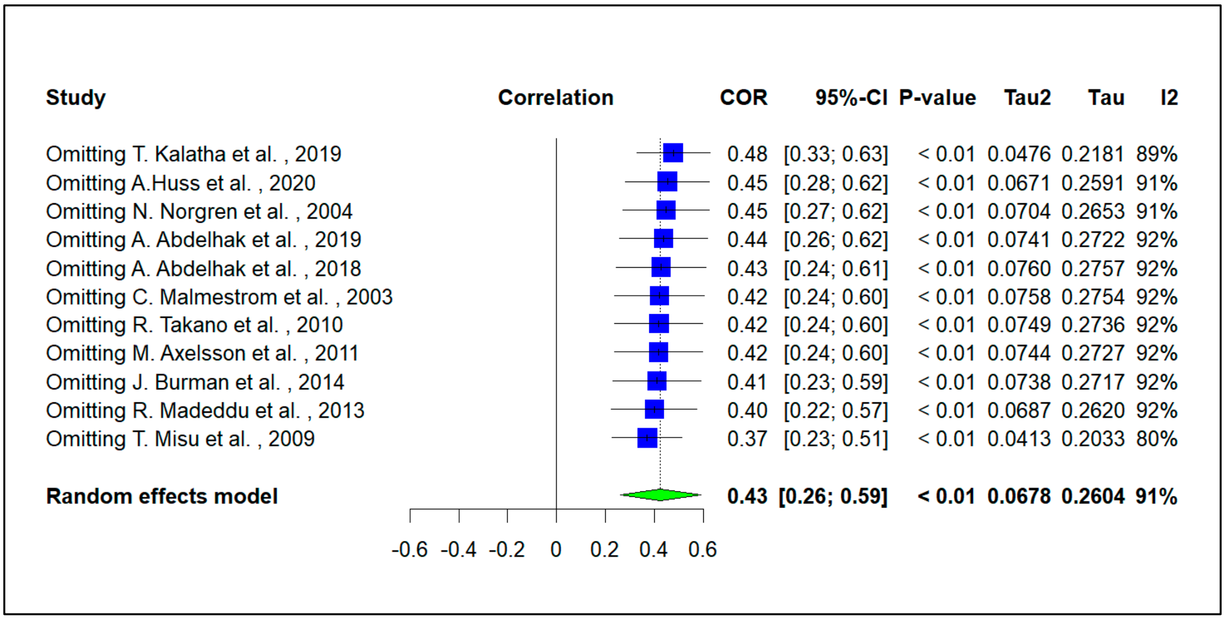

Figure S16. Sensitivity analysis of the meta-analysis of the correlation between cGFAP and EDSS in PwMS.

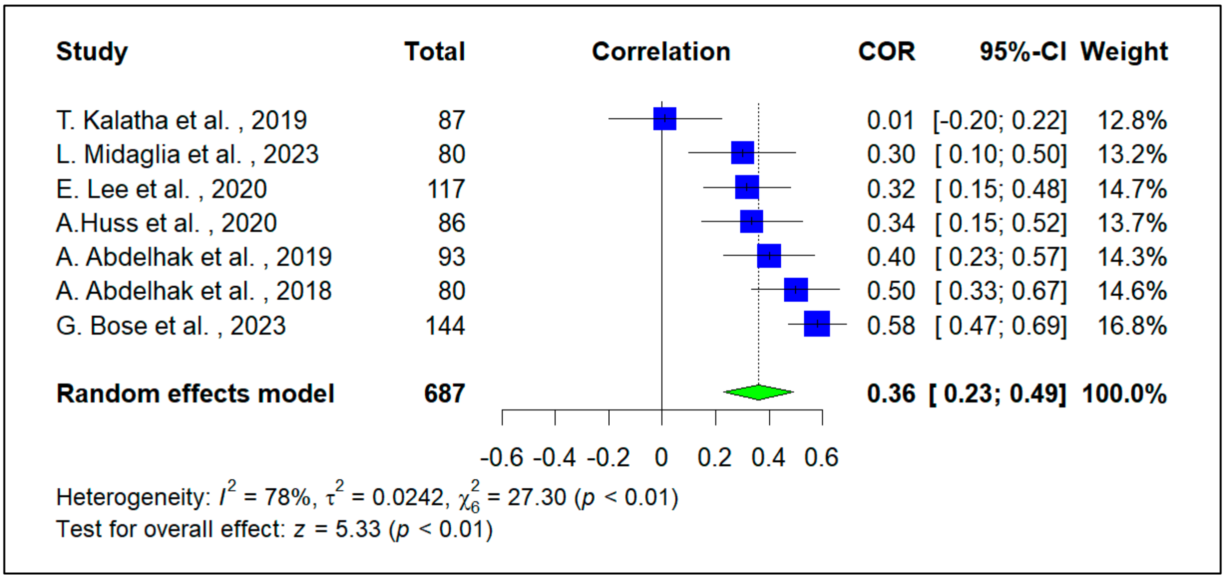

Figure S17. Forest plot of the meta-analysis of the correlation between sGFAP and EDSS in PwMS.

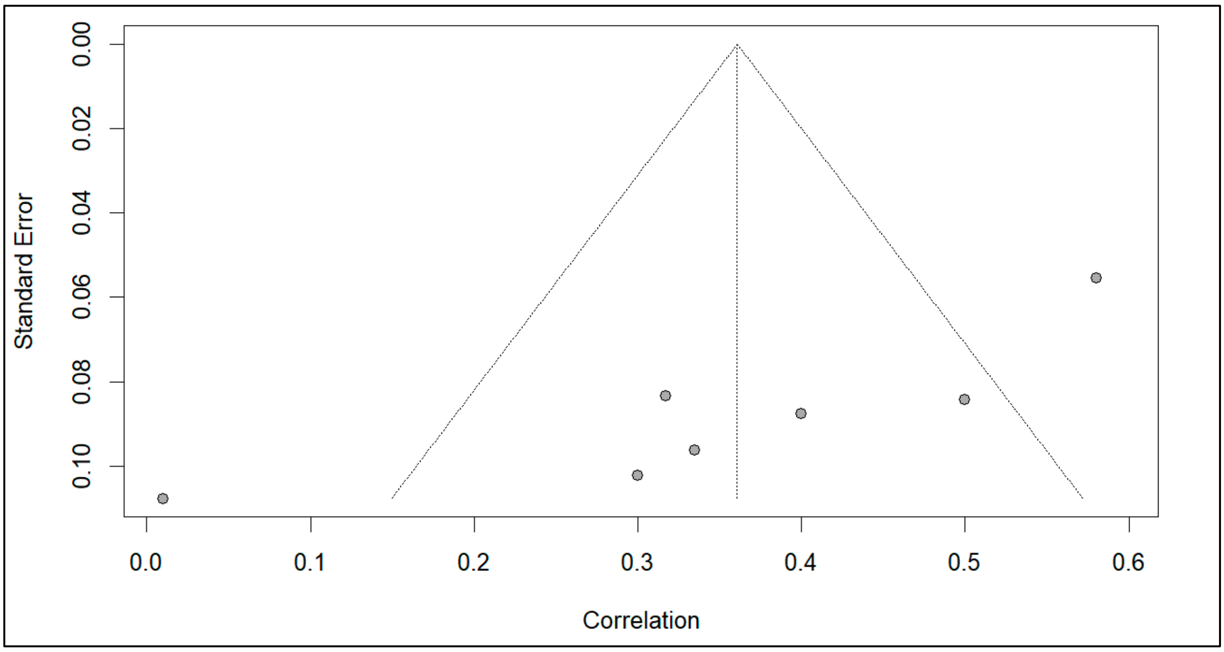

Figure S18. Funnel plot of the meta-analysis of the correlation between sGFAP and EDSS in PwMS.

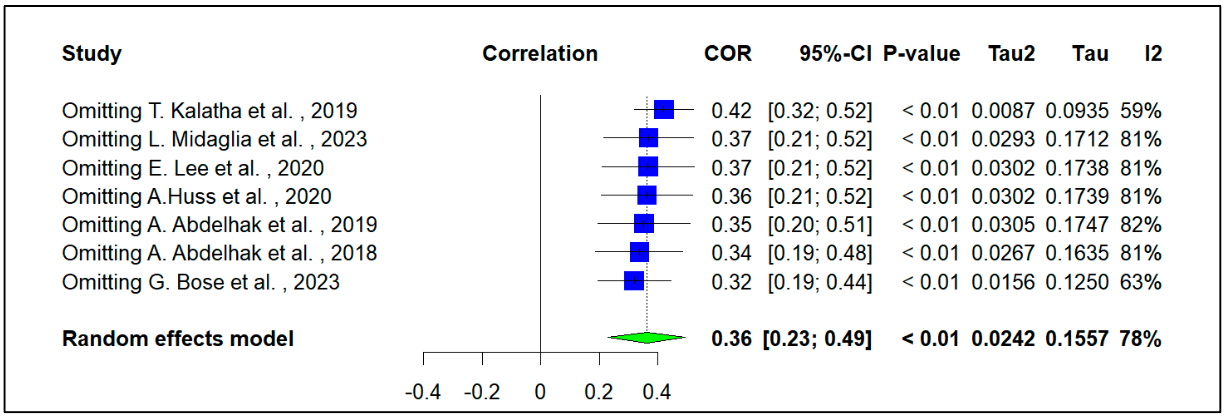

Figure S19. Sensitivity analysis of the meta-analysis of the correlation between sGFAP EDSS in PwMS.

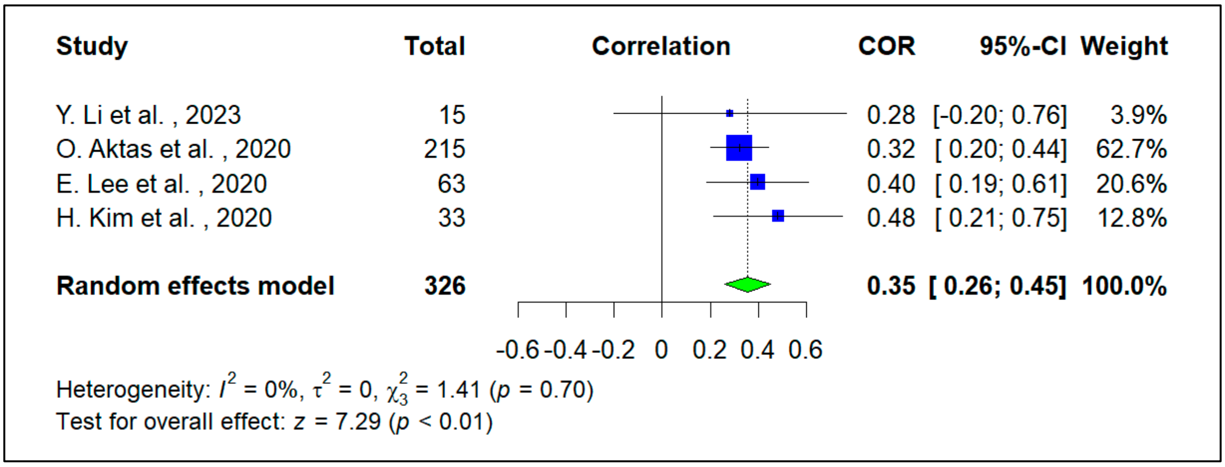

Figure S20. Forest plot of the meta-analysis of the correlation between sGFAP and EDSS in PwNMOSD.

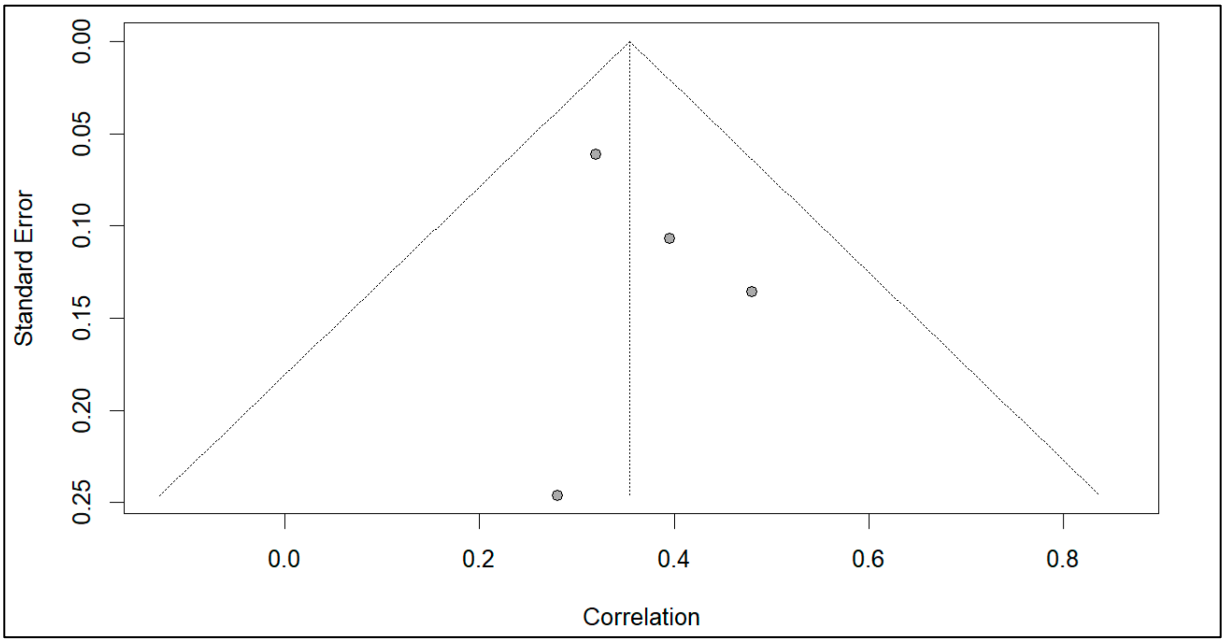

Figure S21. Funnel plot of the meta-analysis of the correlation between EDSS in PwNMOSD.

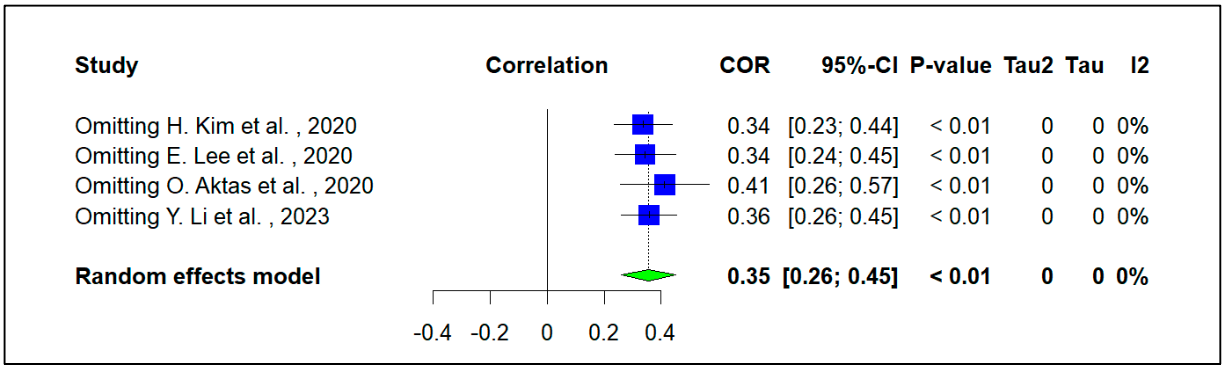

Figure S22. Sensitivity analysis of the meta-analysis of the correlation between sGFAP and EDSS in PwNMOSD.

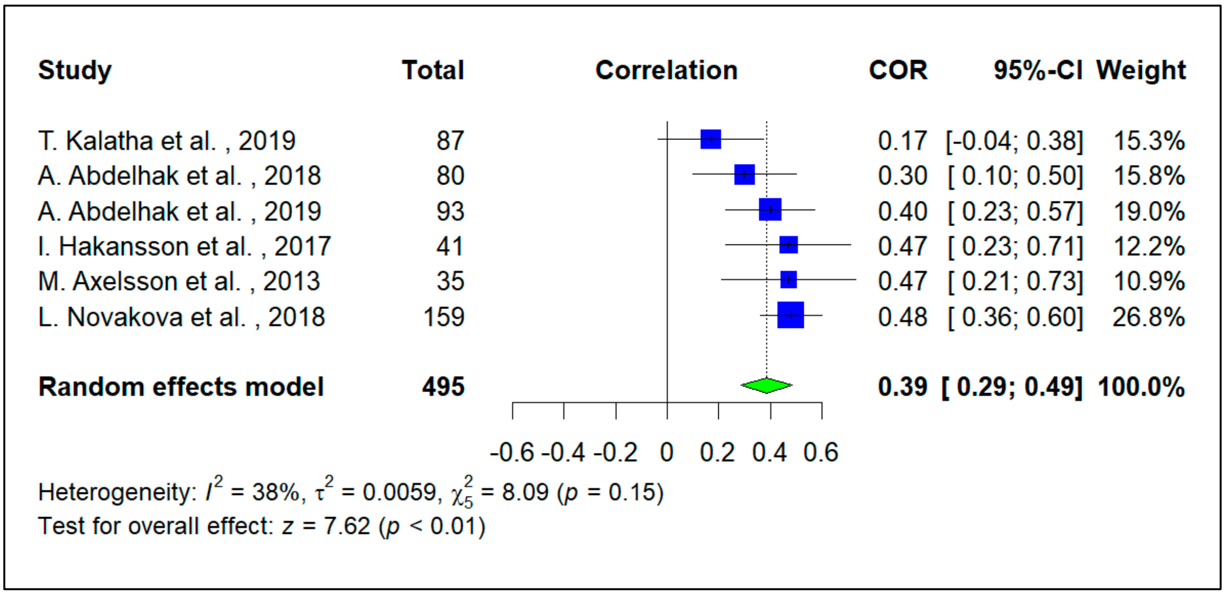

Figure S23. Forest plot of the meta-analysis of the correlation between cGFAP and neurofilament light chain (NfL) in PwMS.

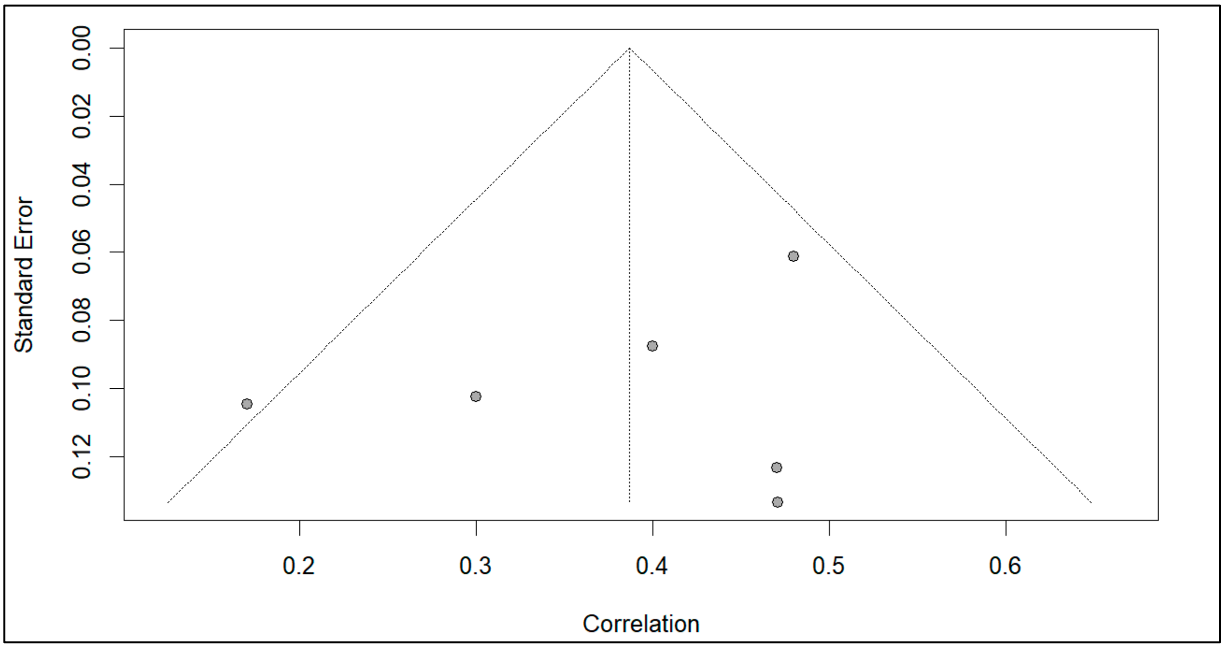

Figure S24. Funnel plot of the meta-analysis of the correlation between cGFAP and NfL in PwMS.

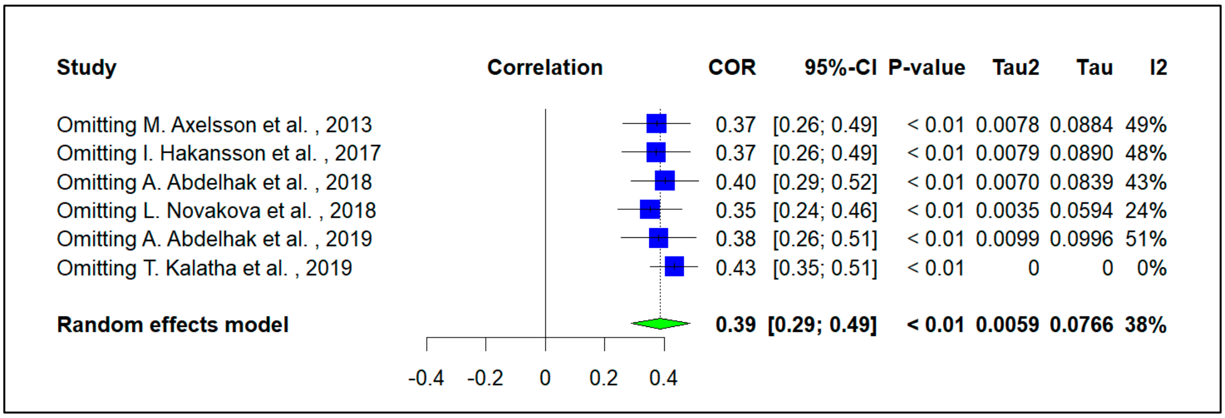

Figure S25. Sensitivity analysis of the meta-analysis of the correlation between cGFAP and NfL in PwMS.

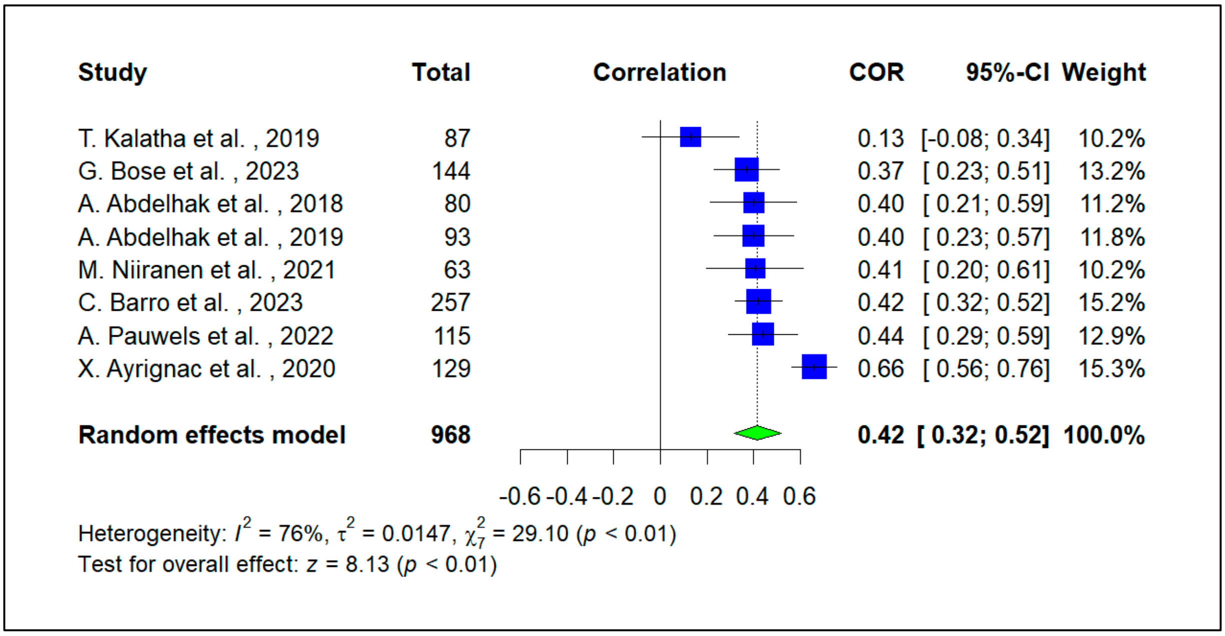

Figure S26. Forest plot of the meta-analysis of the correlation between sGFAP and NfL in PwMS.

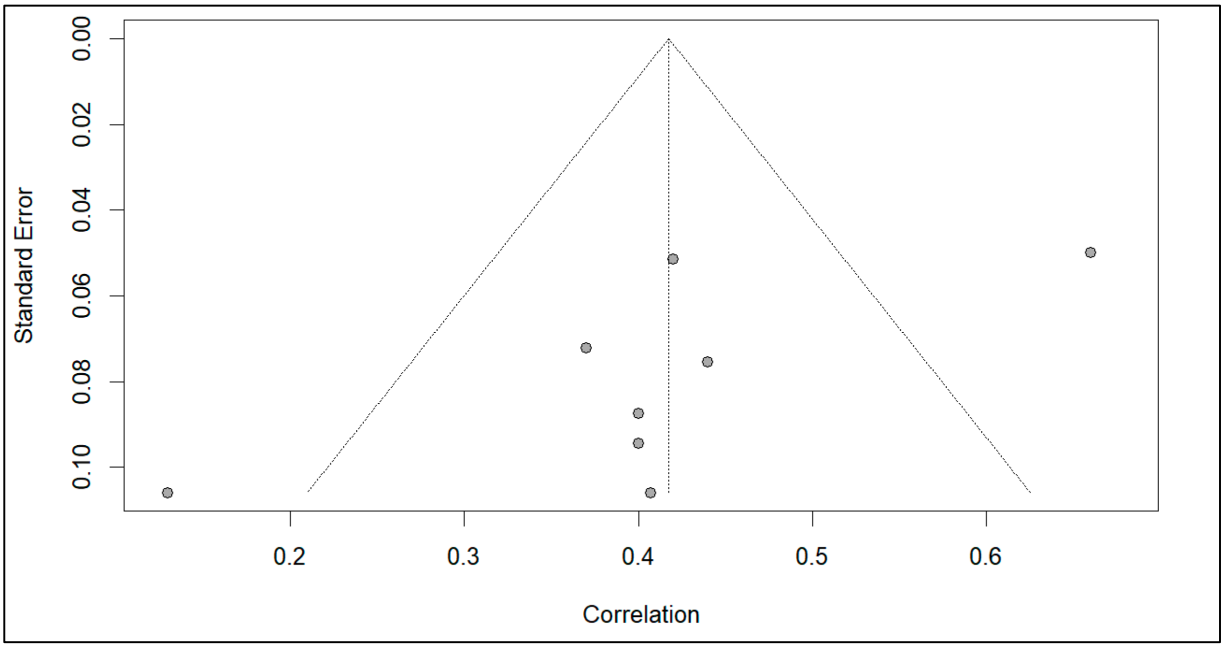

Figure S27. Funnel plot of the meta-analysis of the correlation between NfL in PwMS.

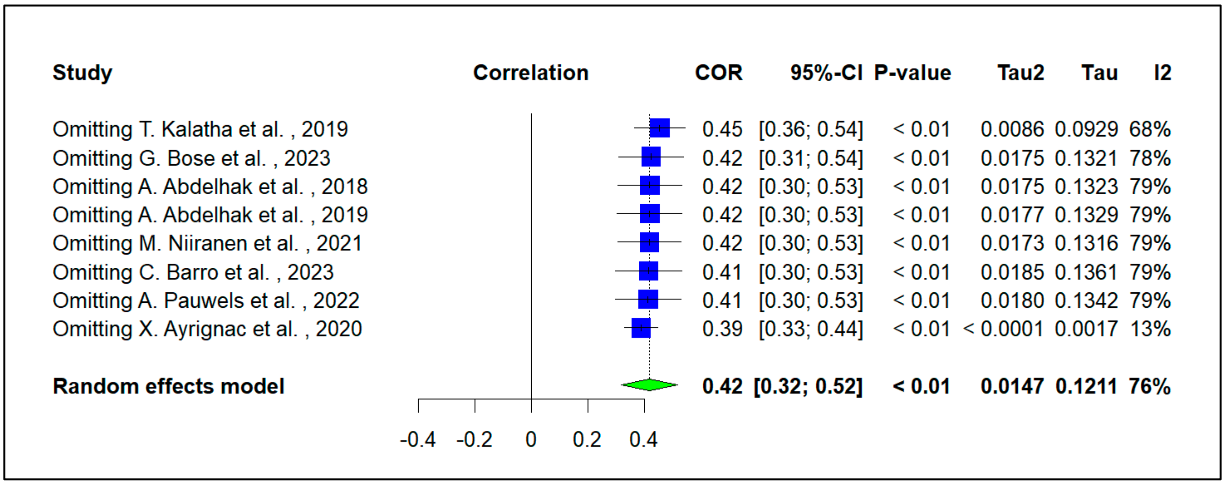

Figure S28. Sensitivity analysis of the meta-analysis of the correlation between sGFAP and NfL in PwMS.

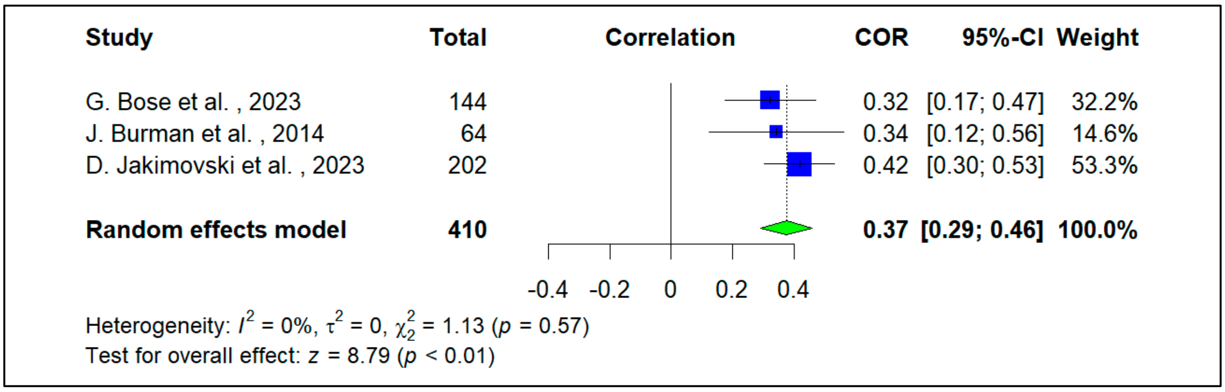

Figure S29. Forest plot of the meta-analysis of the correlation between CSF and serum level of GFAP and T2 lesion volume (T2LV) in PwMS.

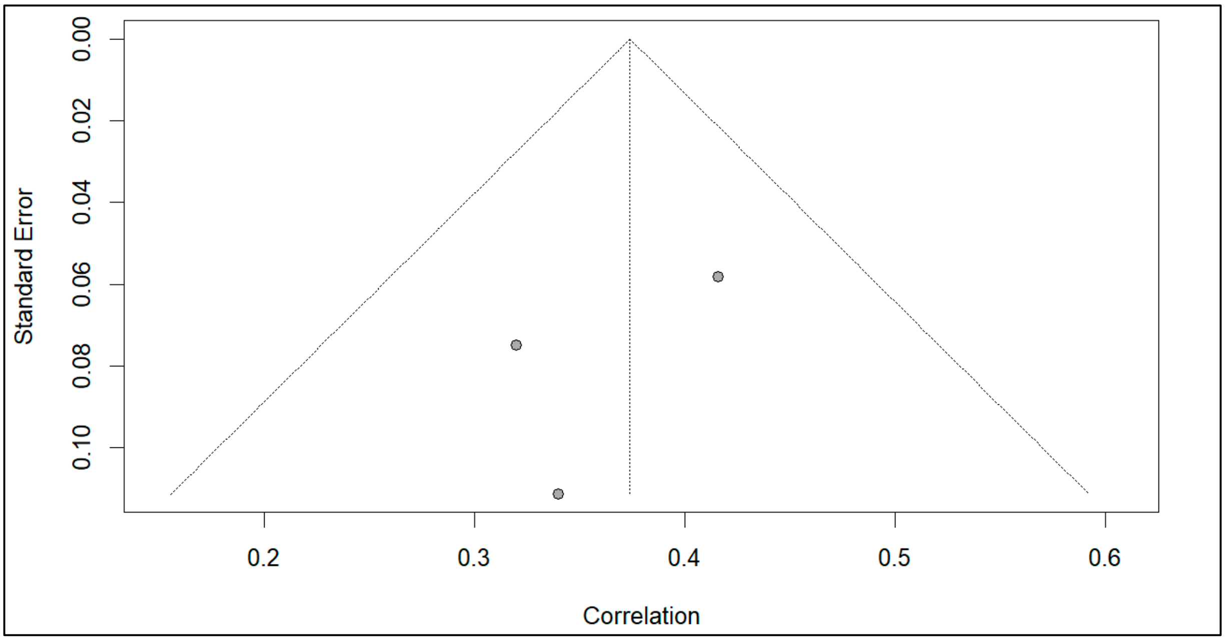

Figure S30. Funnel plot of the meta-analysis of the correlation between CSF and serum level of GFAP and T2LV in PwMS.

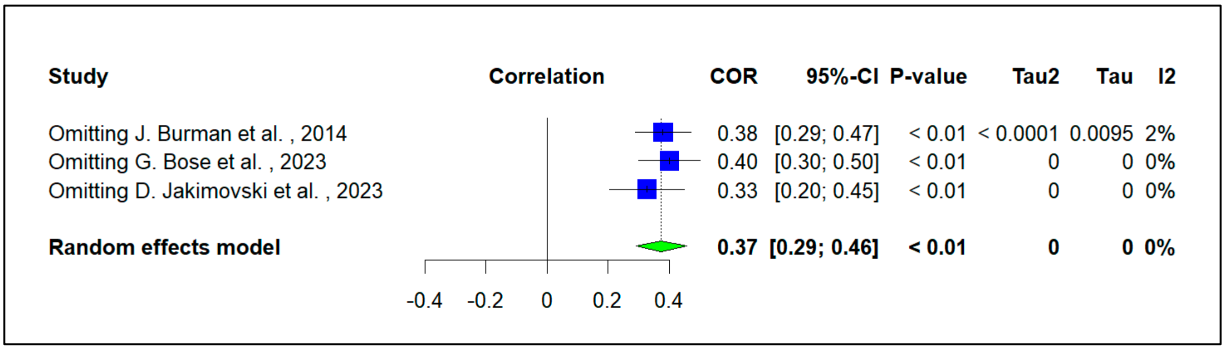

Figure S31. Sensitivity analysis of the meta-analysis of the correlation between CSF and serum level of GFAP and T2LV in PwMS.
